# Gemcitabine exhibits a suppressive effect on pancreatic cancer cell growth by regulating processing of PVT1 to miR1207

**DOI:** 10.1002/1878-0261.12393

**Published:** 2018-10-30

**Authors:** Lei You, Huanyu Wang, Gang Yang, Fangyu Zhao, Jingcheng Zhang, Ziwen Liu, Taiping Zhang, Zhiyong Liang, Changzheng Liu, Yupei Zhao

**Affiliations:** ^1^ Department of General Surgery Peking Union Medical College Hospital Chinese Academy of Medical Sciences Peking Union Medical College Beijing China; ^2^ Department of Pathology Peking Union Medical College Hospital Chinese Academy of Medical Sciences Peking Union Medical College Beijing China; ^3^ Department of Biochemistry and Molecular Biology Institute of Basic Medical Sciences Chinese Academy of Medical Sciences School of Basic Medicine Peking Union Medical College Beijing China; ^4^ MOH Key Laboratory of Systems Biology of Pathogens and Christophe Mérieux Laboratory IPB, CAMS‐Fondation Mérieux Institute of Pathogen Biology (IPB) Chinese Academy of Medical Sciences (CAMS) Peking Union Medical College Beijing China

**Keywords:** gemcitabine chemotherapy, miRNA processing, pancreatic cancer, PVT1

## Abstract

Gemcitabine serves as a first‐line chemotherapy agent for advanced pancreatic cancer (PC). However, the molecular basis by which gemcitabine exerts its effects is not well‐established, and the targeted genetic pathways remain unclear. *Pvt1* oncogene (non‐protein coding) (PVT1) has been reported to be an oncogenic long non‐coding RNA in tumorigenesis. In the present study, we show that the expression of PVT1 is correlated with gemcitabine efficacy in PC therapy. Inhibition of PVT1 led to decreased cell growth in PC cells treated with gemcitabine. We also demonstrate that gemcitabine treatment decreases PVT1 levels and increases its encoded miRNAs, such as the miR‐1207 pair (miR‐1207‐5p/3p). Overexpression of the miR‐1207 pair enhanced the chemosensitivity of cells to gemcitabine, whereas silencing of miR‐1207‐5p/3p to prevent its induction by gemcitabine treatment led to increased cell growth. Mechanistic studies revealed that miR‐1207‐5p and miR‐1207‐3p target the SRC proto‐oncogene (non‐receptor tyrosine kinase) and ras homolog family member A in PC cells, respectively. In particular, we observed that gemcitabine induced Drosha ribonuclease III (Drosha) and DGCR8 microprocessor complex subunit (DGCR8) upregulation and then triggered PVT1 processing. Suppression of Drosha and DGCR8 contributed to a dampened efficacy of gemcitabine, indicating that gemcitabine decreased PVT1 expression by promoting its processing into miRNAs, which in turn resulted in blunted oncogenic signaling in PC cells. Moreover, we demonstrate that gemcitabine chemoresistance was a result of decreased expression of Drosha and DGCR8 in AsPC‐1 cells and tumor cell‐engrafted models. Overall, our findings define a novel mechanism for understanding the efficacy of gemcitabine chemotherapy in PC.

**Abbreviations**
ATCC: American type culture collection
CCK‐8: Cell Counting Kit‐8
DGCR8: DGCR8 microprocessor complex subunit
Drosha: Drosha ribonuclease III
HRP: horseradish peroxidase
IHC: immunohistochemistry
lncRNA: long non‐coding RNA
miRNA: microRNA
MYC: MYC proto‐oncogene, bHLH transcription factor
NS: normal saline
PC: pancreatic cancer
PVT1: *Pvt1* oncogene (non‐protein coding)
qRT‐PCR: quantitative RT‐PCR
RhoA: ras homolog family member A
Scr: scramble
SRC: SRC proto‐oncogene, non‐receptor tyrosine kinase

## 1 Introduction

Pancreatic cancer (PC) is one of the major human cancers with a poor clinical prognosis and over 80% of patients suffering from PC have incurable disease at the time of diagnosis, with an overall survival rate of less than 7% (Seton‐Rogers, 2015; Whitcomb *et al*., 2015; Wolpin *et al*., 2014). Although chemotherapy plays a crucial role in PC treatment, the inherent resistance to currently available chemotherapeutic drugs presents a major challenge (Dolmans *et al*., 2003; Li and O'Reilly, 2015). Gemcitabine is an efficacious first‐line chemotherapy agent for PC treatment (Alderton, 2012; Li and O'Reilly, 2015; de Sousa Cavalcante and Monteiro, 2014). However, the mechanism accounting for gemcitabine resistance in PC treatment is not well‐established and how to improve its chemosensitivity in PC cells needs to be investigated further.

*Pvt1* oncogene (non‐protein coding) (PVT1) is a large locus that is adjacent to the *c‐myc* on human chromosome 8q24 (Huppi *et al*., 2012). Recent studies indicate that co‐amplification of human MYC proto‐oncogene, bHLH transcription factor (MYC) and PVT1 is correlated in primary human tumors and that the gain of PVT1 long non‐coding RNA (lncRNA) expression is required for high MYC protein levels in 8q24‐amplified human cancer cells (Iwakawa *et al*., 2013; Tseng *et al*., 2014; Wolpin *et al*., 2014). Our previous study also validates PVT1 (in humans, PVT1, for plasmacytoma variant translocation) as a critical regulator of gemcitabine chemosensitivity in PC cells using a genome‐wide and *piggyBac* transposon‐based genetic screening platform (You *et al*., 2011). These findings clearly indicate that PVT1 deregulation is involved in tumorigenesis and progression. With no protein product or consensus lncRNA, the functional implication of PVT1 processing has been difficult to discern as being attributed to its encoded microRNAs (miRNAs) (miR‐1204‐1208 family) (Beck‐Engeser *et al*., 2008). Increasing evidence has indicated that several miRNAs are involved in regulating gemcitabine chemosensitivity in PC cells. For example, miR‐429‐induced PDCD4 and miR‐10a‐induced TFAP2C signaling pathways are reported as potential targets in the improvement of gemcitabine therapy (Xiong *et al*., 2018; Yu *et al*., 2017). Inhibition of miR‐21 or ‐221 sensitized gemcitabine efficacy by regulating the cell cycle and inducing apoptosis (Park *et al*., 2009). However, the roles of *pvt1*‐encoded miRNAs are not well‐established in PC cells with gemcitabine treatment. Furthermore, whether the processing of PVT1 switch to mature miRNAs is involved in gemcitabine chemotherapy remains unclear.

In the present study, we identified PVT1 as a potential chemotherapeutic target of gemcitabine and demonstrated that gemcitabine triggered PVT1 processing into miRNAs by regulating the function of the microprocessor in PC cells. Moreover, we found that the expression of the miR‐1207 pair was upregulated and then the targeted oncogenic signaling was suppressed in PC cells upon gemcitabine treatment.

## 2 Materials and methods

### 2.1 Clinical specimens

Tissues were collected as described previously (Liu *et al*., 2010). In total, 10 patients with PC from Peking Union Medical College Hospital were included in the present study, and fresh samples were snap frozen in liquid nitrogen immediately after resection and stored at −80 °C. The study was approved by the ethical board of hospital and the ethical board of the Institute of Basic Medical Sciences, Chinese Academy of Medical Sciences. The experiments were undertaken with the understanding and written consent of each subject and the study methodologies conformed with the standards set by the Declaration of Helsinki. The tissue samples were histologically confirmed by staining with hematoxylin and eosin. The patient characteristics are provided in Table S1.

### 2.2 Cell culture and drug treatment

The 293T cell line and the tested PC cell lines (MIA PaCa‐2, Su.86.86, Capan‐1, PANC‐1, SW1990, BxPC‐3 and AsPC‐1) were obtained from the American Type Culture Collection (Manassas, VA, USA) and grown in Dulbecco's modified Eagle's medium with 10% fetal bovine serum (HyClone, South Logan, UT, USA) at 37 °C in a 5% CO_2_ cell culture incubator. The PC cell line and the gemcitabine‐resistant PC cell lines were purchased from the Shanghai Cell Bank, Chinese Academy of Sciences (Shanghai, China) and cultured under standard conditions. Cell lines were tested 1 month before the experiment with respect to microscopic morphology, growth curve analysis and mycoplasma detection in accordance with the ATCC cell line verification test recommendations. PC cells were seeded overnight and then treated with 10–50 μg·mL^−1^ gemcitabine (Eli Lilly & Co., Indianapolis, IN, USA) for 72 h. Normal saline (NS) served as a control.

### 2.3 RNA isolation and quantitative RT‐PCR (qRT‐PCR) analysis

Total RNA was extracted from cells and tissues using Trizol (Invitrogen, Carlsbad, CA, USA) in accordance with the manufacturer′s instructions. The RNA was quantified by absorbance at 260 nm. To assess the levels of miRNAs, qRT‐PCR analysis was conducted by using Taqman probes (Invitrogen) in an IQ5 Q‐PCR system (Bio‐Rad, Hercules, CA, USA) in accordance with the manufacturer′s instruction. Primers for qRT‐PCR are shown in Tables S2 and S3.

### 2.4 Quantification of protein

Western blotting of proteins was performed as described previously (Liu *et al*., 2010). The antibodies included those against SRC proto‐oncogene, non‐receptor tyrosine kinase (SRC), ras homolog family member A (RhoA), Drosha ribonuclease III (Drosha), DGCR8 microprocessor complex subunit (DGCR8) and GAPDH (Drosha and DGCR8, from CST, Danvers, MA, USA; SRC, RhoA and GAPDH from Abcam, Cambridge, MA, USA).

### 2.5 Cell proliferation, apoptosis and cell cycle analysis

To measure the effects on cellular proliferation rates, PC cells were incubated in 10% Cell Counting Kit‐8 (CCK‐8; Dojindo, Kumamoto, Japan) diluted in normal culture media at 37 °C until visual color conversion appears. Proliferation rates were determined at 12, 24, 36, 48, 60 and 72 h post‐transfection and quantification was performed on a microtiter plate reader (Spectra Rainbow; Tecan, Männedorf, Switzerland) in accordance with the manufacturer's instructions.

Cell apoptosis assay was performed on BxPC‐3, PANC‐1 and AsPC‐1 PC cells by using the Annexin V‐FITC Apoptosis Detection kit I (BD Biosciences, San Jose, CA, USA) in accordance with the manufacturer's instructions and data were analyzed using a FACS Calibur Flow Cytometer (Becton‐Dickinson Biosciences, Franklin Lakes, NJ, USA).

Cell cycle analysis was performed for BxPC‐3, PANC‐1 and AsPC‐1 PC cells. Data were collected and processed using modfit cell cycle analysis software (Verity, Topsham, ME, UK).

### 2.6 Constructs, reagents and assays

The 3′ UTR of the human SRC and RhoA mRNA was cloned into the *Not1* and *Xba*1 sites of pRL‐TK (Promega, Madison, WI, USA). The AKT1 and c‐Jun sequences were mutated using a QuickChange Site‐Directed Mutagenesis kit (Stratagene, La Jolla, CA, USA). The primer sequences are shown in Table S3. miRNA mimics and inhibitors specifying miR‐1204, miR‐1207‐5p/3p, miR‐1208 and control miRNA mimic/inhibitor were obtained from Dharmacon (Lafayette, CO, USA).

The 293T cells were seeded onto 24‐well plates (1 × 10^5^ cells·well^−1^) the day before transfection was performed. Cells (approximately 70% confluent) were transfected with a pRL‐TK luciferase reporter (50 ng·well^−1^), a pGL3‐control firefly luciferase (10 ng·well^−1^) or a miR‐1207‐5p/3p mimic (50 nm). All transfections were performed in triplicate with Lipofectamine 2000 (Invitrogen). Cell lysates were prepared with Passive Lysis Buffer (Promega) 48 h after transfection, and luciferase activities were measured with the Dual Luciferase Reporter Assay (Promega).

### 2.7 Immunohistochemistry

Immunohistochemistry (IHC) was conducted to measure SRC and RhoA expression as described previously (Du *et al*., 2017). In brief, paraffin‐embedded tissue was pretreated at 65 °C for 2 h, followed by deparaffinization using standard procedures. Antigen retrieval was performed in antigen retrieval solution before application of primary antibodies. Slides were then incubated for 2 h at room temperature with the secondary antibody conjugated with horseradish peroxidase (HRP). HRP activity was detected using the Histostain‐plus kit (Invitrogen) in accordance with the manufacturer's instructions. Evaluation of IHC staining was performed as described previously (Waltregny *et al*., 1998). In brief, IHC‐stained sections were examined using a Vanox‐T AH‐2 light microscope (Olympus, Tokyo, Japan). The same magnification (microscope objective) was used to record all images in a particular series. Both the field limiting and contrast apertures were kept at the fully open position during (digital) photography to avoid any variability in reproducing aperture settings. Furthermore, the IHC‐stained sections were reviewed by two independent observers unbiased without the knowledge of clinical outcome.

### 2.8 PC cell‐engrafted tumor mouse model

All animal procedures were performed as described previously (Du *et al*., 2017; He *et al*., 2015). The animal experiments were conducted in accordance with the national Animal Experimentation guidelines (D.L.116/92) upon approval of the experimental protocol by the Institutional Animal Experimentation Committee of Peking Union Medical College. For the xenograft assay, 6‐week‐old female nude mice (BALB/c‐nude) were used to examine tumorigenicity. BxPC‐3 and AsPC‐1 cells were propagated [6 × 10^6^ cells saline/Matrigel (BD Pharmigen, San Jose, CA, USA), 1 : 1 v/v] and inoculated s.c. into the dorsal flanks of mice. The size of the tumors was measured twice a week using calipers, and tumor volumes were calculated using the formula: Л/6 × *d* ^2^ × *D*, then removed and weighed 7 weeks after tumor cell injection. Treatments were started after tumor reached approximately 100 mm^3^. Gemcitabine was administered twice a week at 50 mg·kg^−1^ of body weight via intraperitoneal injection for 3 weeks. Control mice received physiological saline only, according to the same schedule.

### 2.9 Statistical analysis

Each experiment was repeated at least three times. Student's *t* test (two‐tailed) was performed and three‐group data were analyzed using one‐way analysis of variance. All statistical analyses were performed using spss, version 16.0 software (SPSS Inc., Chicago, IL, USA). *P* < 0.05 was considered statistically significant.

## 3 Results

### 3.1 PVT1 inhibition leads to improved efficacy of gemcitabine in PC cells

**Figure 1:**
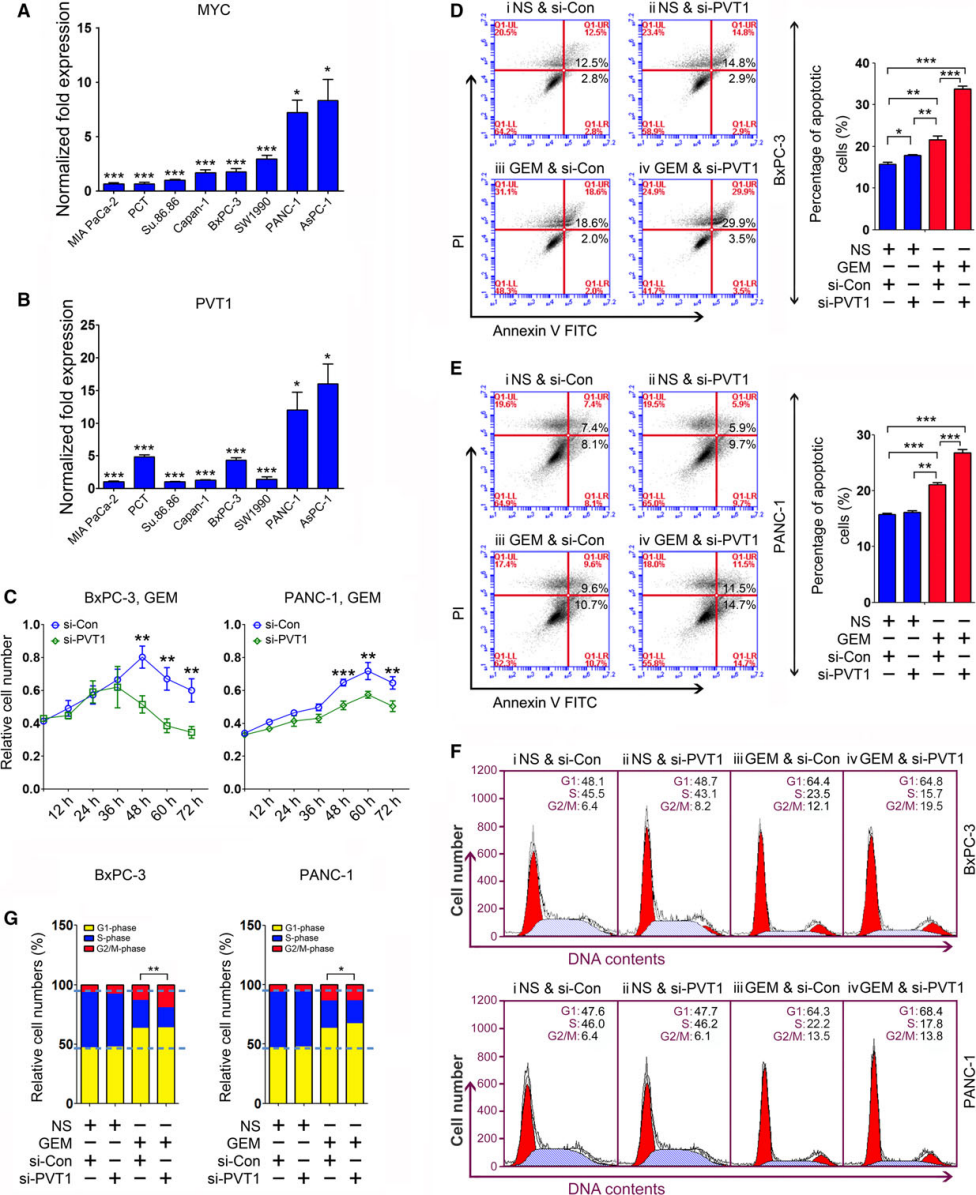
The efficacy of gemcitabine chemotherapy is improved in PC cells by PVT1 inhibition. (A,B) Expression of MYC (A) and PVT1 (B) in PC cells was determined by qRT‐PCR. Data were normalized to GAPDH. (C) Cell growth was analyzed in BxPC‐3 (left) and PANC‐1 (right) cells with gemcitabine treatment by using a CCK‐8 assay at 12 h intervals up to 72 h. (D,E) Apoptosis assays were used to measure programmed cell death in BxPC‐3 (D) and PANC‐1 (E) cells upon PVT1 inhibition and gemcitabine treatment. Normalization of the apoptotic cells is shown on the right in (D) and (E). (F,G) Cell cycle analyses were performed in BxPC‐3 (upper) and PANC‐1 (lower) cells (F) and normalization of cell numbers at G1‐, S‐ and G2/M‐phase is shown in (G). *P <* *0.05, **P <* *0.01, ***P <* *0.001. Mean ± SD values were calculated from triplicate samples. *P* values were based on Student's *t* test unless otherwise indicated.

Previous work indicated that PVT1 overexpression resulted in a decreased efficacy of gemcitabine in PC cells (You *et al*., 2011). In the present study, we inhibited PVT1 with a specific siRNA (si‐PVT1) to further investigate the correlation between PVT1 and gemcitabine chemosensitivity. Thus, we determined the expression of PVT1 in several PC cell lines. qRT‐PCR analysis revealed that PVT1 had an expressional profile similar to that of MYC in these examined cells (Fig. 1A,B). Next, BxPC‐3 and PANC‐1 cells were transiently transfected with si‐PVT1 after the half maximal inhibitory concentration value was determined (Fig. S1A,B). The cell proliferation rate was analyzed by the CCK‐8 assay, which showed that PVT1 inhibition led to decreased cell growth in these two cells with gemcitabine treatment (Fig. 1C). No significant difference was observed between si‐PVT1‐ and si‐Con‐treated cells upon NS treatment (Fig. S1C). To further investigate the impact of PVT1 on cell growth, apoptosis and the cell cycle were analyzed, revealing that PVT1 inhibition resulted in increased apoptosis in BxPC‐3 and PANC‐1 cells upon gemcitabine treatment (Fig. 1D,E). It was also observed that si‐PVT1 treatment led to decreased numbers of cells in S‐phase in these two gemcitabine‐treated cells (Figs 1F,G and S1D,E).

Altogether, these data indicate that PVT1 inhibition contributes to an improved gemcitabine chemosensitivity in PC cells.

### 3.2 PVT1 switch to the miR‐1207 pair is involved in regulating the gemcitabine efficacy in PC cells

**Figure 2:**
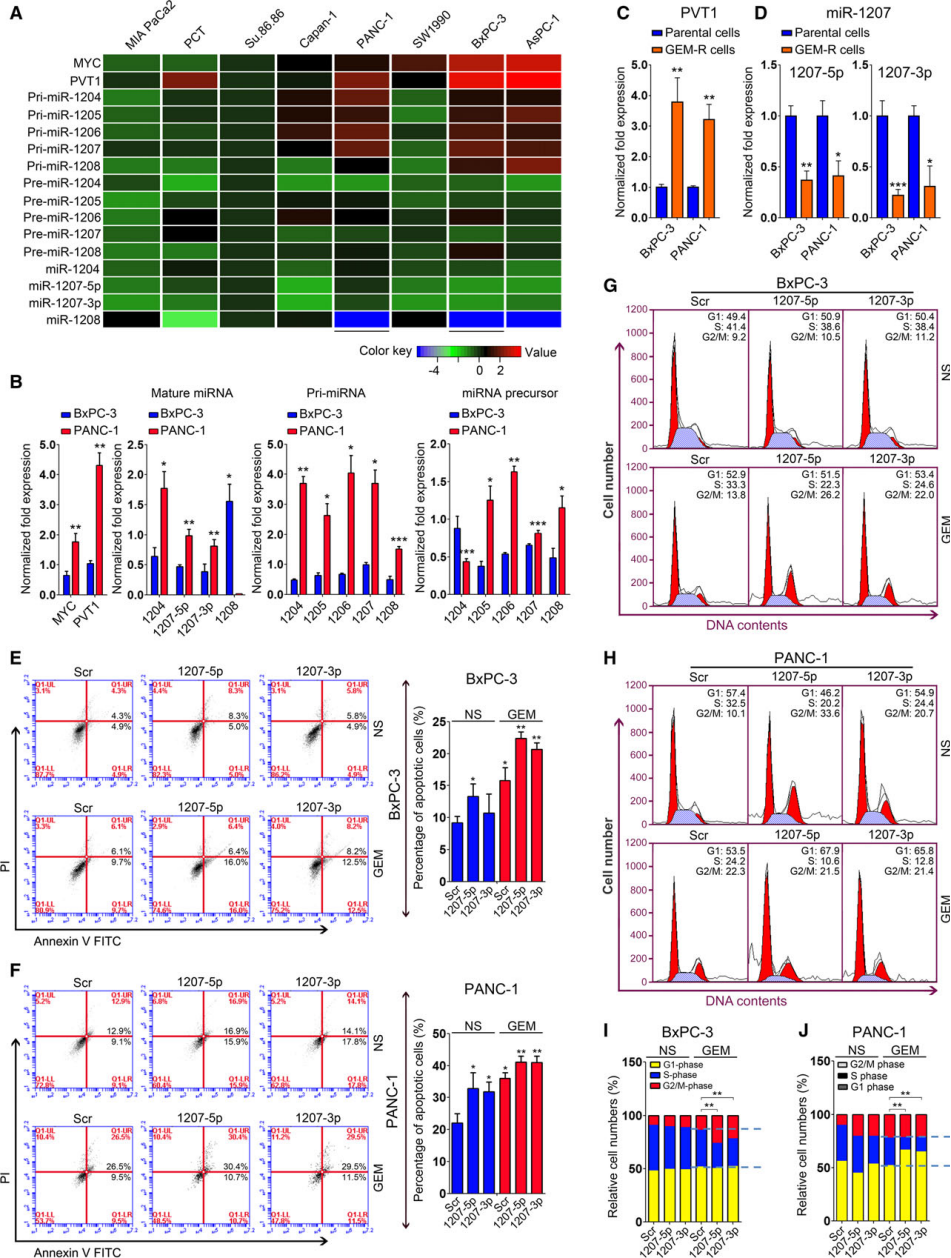
PVT1 switch to mature miRNAs is involved in the regulation of gemcitabine efficacy in PC cells. (A,B) qRT‐PCR analysis was conducted to determine the expression of MYC and PVT1 transcripts in several PC cell lines, including BxPC‐3 and PANC‐1 (B). GAPDH was used as a loading control to detect the expression of MYC, PVT1 and pri‐miRNAs. U6 snRNA served as a loading control for the detection of miRNA precursors and mature miRNAs. (C,D) Expression of PVT1 and miR‐1207 pair was determined in gemcitabine‐resistant BxPC‐3 and PANC‐1 cells using qRT‐PCR analysis. GAPDH was used as a loading control to detect the expression of PVT1 and U6 snRNA served as a loading control for the detection of miR‐1207‐5p/3p. (E,F) Apoptosis assays were performed in BxPC‐3 (E) and PANC‐1 (F) cells with the transfection of miR‐1207 mimics and gemcitabine treatment. Normalization of the apoptotic cells is shown on the right. (G–J) Cell cycle analyses were conducted in BxPC‐3 (G) and PANC‐1 (H) ells, and normalization of cell numbers at G1‐, S‐ and G2/M‐phase are shown in (I) and (J). *P <* *0.05, **P <* *0.01, ***P <* *0.001. Mean ± SD values were calculated from triplicate samples. *P* values were based on Student's *t* test unless otherwise indicated.

A previous study indicated that the *pvt1* locus encodes several miRNAs, including miR‐1204, miR‐1205, miR‐1206, the miR‐1207 pair (miR‐1207‐5p/3p) and miR‐1208 (Beck‐Engeser *et al*., 2008). Although our above findings demonstrated that gemcitabine chemosensitivity was improved in PC cells with decreased PVT1 levels, it remained unclear whether these miRNAs were correlated with gemcitabine efficacy. To this end, we investigated the expressional profile of miR‐1204‐1208 family members in PC cells. qRT‐PCR analysis revealed that the expression of primary miRNAs, miRNA precursors and mature miRNAs was upregulated in PC cells with increased PVT1 levels, except for mature miR‐1208 (Fig. 2A,B). Mature miR‐1205 and miR‐1206 were not detected in the PC cells that were examined (data not shown). We next investigated the role of these miRNAs in PC cell growth. The corresponding miRNA mimics of miR‐1204, the miR‐1207 pair or miR‐1208 were transfected into BxPC‐3 and PANC‐1 cells (Fig. S2A,B) and a CCK‐8 assay was performed, which indicated that overexpression of miR‐1204, the miR‐1207 miRNA pair and miR‐1208 led to dampened cell proliferation (Figs S3 and S4A). No obvious difference was observed between scramble (Scr)‐ and miR‐1208‐treated BxPC‐3 cells (Fig. S4B). These data suggest a regulatory role for the *pvt1* locus and a potential relationship between the miR‐1204‐1208 family and PVT1 function.

Furthermore, we explored the function of miR‐1204 and the miR‐1207 pair in PC cells upon gemcitabine treatment. Cell growth analysis revealed that enforced expression of miR‐1204 and the miR‐1207 pair resulted in reduced cell proliferation in BxPC‐3 and PANC‐1 cells treated with gemcitabine (Fig. S3). Based on these findings, we considered whether PVT1 switch to cell growth suppressive miRNAs (e.g. miR‐1207‐5p and miR‐1207‐3p) was involved in the regulation of gemcitabine effect in PC cells. To test this idea, the expression of PVT1 and the miR‐1207 pair was determined in BxPC‐3, PANC‐1 and pair‐matched gemcitabine‐resistant cells. We found that the expression of PVT1 was increased, whereas the miR‐1207 pair demonstrated downregulation in gemcitabine‐resistant cells compared to the parental BxPC‐3 and PANC‐1 cells (Fig. 2C,D).

Altogether, these data suggest that the process of PVT1 into the miR‐1207 pair in PC cells is correlated with the regulation of gemcitabine chemosensitivity.

### 3.3 Overexpression of the miR‐1207 pair improves gemcitabine efficacy in PC cells

We further addressed the impact of the miR‐1207 pair on cell growth via apoptosis and cell cycle analyses. Thus, we transfected miR‐1207‐5p or miR‐1207‐3p mimic into BxPC‐3 and PANC‐1 cells. The subsequent apoptosis assay revealed that overexpression of the miR‐1207 pair led to increased apoptosis upon gemcitabine treatment in BxPC‐3 cells (Fig. 2E). Similar results were observed in PANC‐1 cells (Fig. 2F). We also conducted cell cycle analyses in these two cells with overexpression of the miR‐1207 pair. In NS‐treated cells, no significant difference was noted between Scr‐ and miR‐1207 pair‐treated PC cells with respect to the number of cells in S‐phase. However, we observed a remarkable decrease in the number of cells in S‐phase in both BxPC‐3 (Figs 2G,I and S5A) and PANC‐1 cells upon gemcitabine treatment (Figs 2H,J and S5B).

Taken together, our data demonstrate that increased levels of the miR‐1207 pair lead to improved gemcitabine efficacy in PC cells.

### 3.4 miR‐1207‐5p targets endogenous SRC

**Figure 3:**
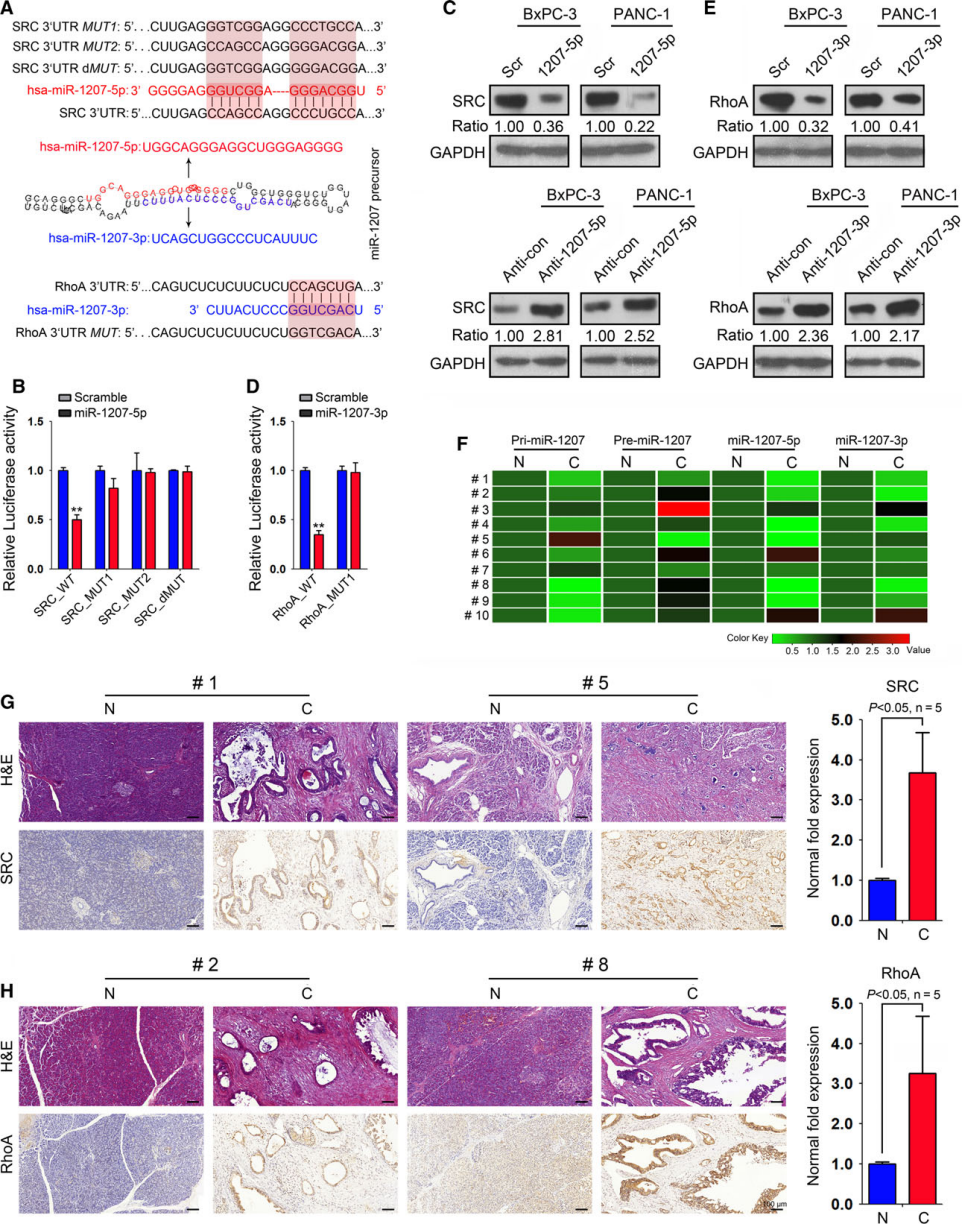
miR‐1207‐5p and 1207‐3p target SRC and RhoA in PC cells. (A) Sequences present in the 3′ UTR of *src* and *RhoA* that are targeted by the miR‐1207 pair. The sequences shaded in gray represent mutations of the miR‐1207‐matched seed sequence. (B) miR‐1207‐5p expression decreased the luciferase activity when linked to the segment containing the target sequence within the 3′ UTR of the *src* mRNA. Mutation of the seed sequence abolished miR‐1207‐5p‐dependent repression. (C) Expression of SRC was evaluated in BxPC‐3 and PANC‐1 cells after miR‐1207‐5p mimic or miR‐1207‐5p inhibitor (anti‐1207‐5p) treatment, as measured by immunoblotting. (D) miR‐1207‐3p expression decreased the luciferase activity when linked to the segment containing the target sequence within the 3′ UTR of the *RhoA* mRNA. Mutation of the seed sequence abolished miR‐1207‐3p‐dependent repression. (E) Expression of RhoA was evaluated in BxPC‐3 and PANC‐1 cells upon miR‐1207‐3p mimic or anti‐1207‐3p treatment, as measured by immunoblotting. GAPDH served as a loading control, and the data were normalized to Scr‐ or anti‐con‐treated PC cells. The numbers below the panels represent the normalized protein expression levels. (F) qRT‐PCR analysis was conducted to evaluate the expression of PVT1, pri‐miR‐1207, pre‐miR‐1207 and mature miR‐1207‐5p/3p in 10 PC tissues (N, matched normal tissues; C, PC tissues). (G,H) Representative images of SRC (G) and RhoA (H) IHC analysis in selected PC samples (SRC, #1 and #5; RhoA, #2 and #8). Magnification is 20×. Scale bars = 100 μm. The fold change was normalized to the matched normal control (N). *P <* *0.05, **P <* *0.01, ***P <* *0.001. Mean ± SD values were calculated from triplicate samples. *P* values were based on Student's *t* test unless otherwise indicated.

To identify the mRNA targets of miR‐1207‐5p that are relevant to gemcitabine chemosensitivity, the miRanda miRNA target prediction program was interrogated, which predicted that human SRC is targeted by miR‐1207‐5p in two regions of its 3′ UTR (Fig. 3A). Transfection of the *src*‐3′ UTR‐luciferase reporter in combination with miR‐1207‐5p mimic in 293T cells indicated that miR‐1207‐5p repressed the luciferase activity of this reporter. Moreover, mutation of miR‐1207‐5p sites abrogated the reduction in luciferase activity (Fig. 3B). Furthermore, elevating miR‐1207‐5p in BxPC‐3 and PANC‐1 cells reduced SRC protein levels, and inhibition of this miRNA led to an increased expression of SRC, as indicated by immunoblotting analysis (Fig. 3C). These data indicate that miR‐1207‐5p regulates the cellular levels of SRC by binding its complementary sites. We also explored the correlation between miR‐1207‐5p and SRC expression in PC tissue specimens. qRT‐PCR and IHC analyses were conducted to evaluate the expression of miR‐1207‐5p and SRC in selected PC samples, which revealed that tumors with decreased miR‐1207‐5p levels demonstrated an elevated expression of SRC (Fig. 3F,G).

Taken together, these findings demonstrate that SRC is a *bona fide* target of miR‐1207‐5p in PC cells.

### 3.5 RhoA is a direct effector of miR‐1207‐3p

Human RhoA is predicted to be targeted by miR‐1207‐3p, and the 3′ UTR of *RhoA* contains a sequence that matches the 7‐mer seed sequence contained in miR‐1207‐3p (Fig. 3A). To validate RhoA as a direct effector of miR‐1207‐3p, we cloned the miR‐1207‐3p predicted target sequence within the RhoA 3′ UTR downstream of a Renilla luciferase reporter gene. We next co‐transfected this reporter plasmid with either a scrambled sequence or a miR‐1207‐3p mimic in 293T cells. The luciferase activity was detected, which indicated a marked repression of luciferase activity for these target sites compared to the control. We further performed target site mutagenesis and observed that mutation of miR‐1207‐3p sites completely abrogated this reduction in luciferase activity, indicating the specificity of the interaction between miR‐1207‐3p and its target regions (Fig. 3D). We next conducted immunoblotting analysis and observed that miR‐1207‐3p overexpression resulted in a significant decrease of RhoA protein levels compared to controls (Fig. 3E). We also noted that inhibition of miR‐1207‐3p led to an increased expression of RhoA (Fig. 3E). Moreover, an inverse association between miR‐1207‐3p and RhoA expression was observed in selected PC tissues (Fig. 3F,H).

Altogether, these data indicate that RhoA is a direct target of miR‐1207‐3p in PC cells.

### 3.6 Inhibition of the miR‐1207 pair decreases gemcitabine chemosensitivity in PC cells

**Figure 4:**
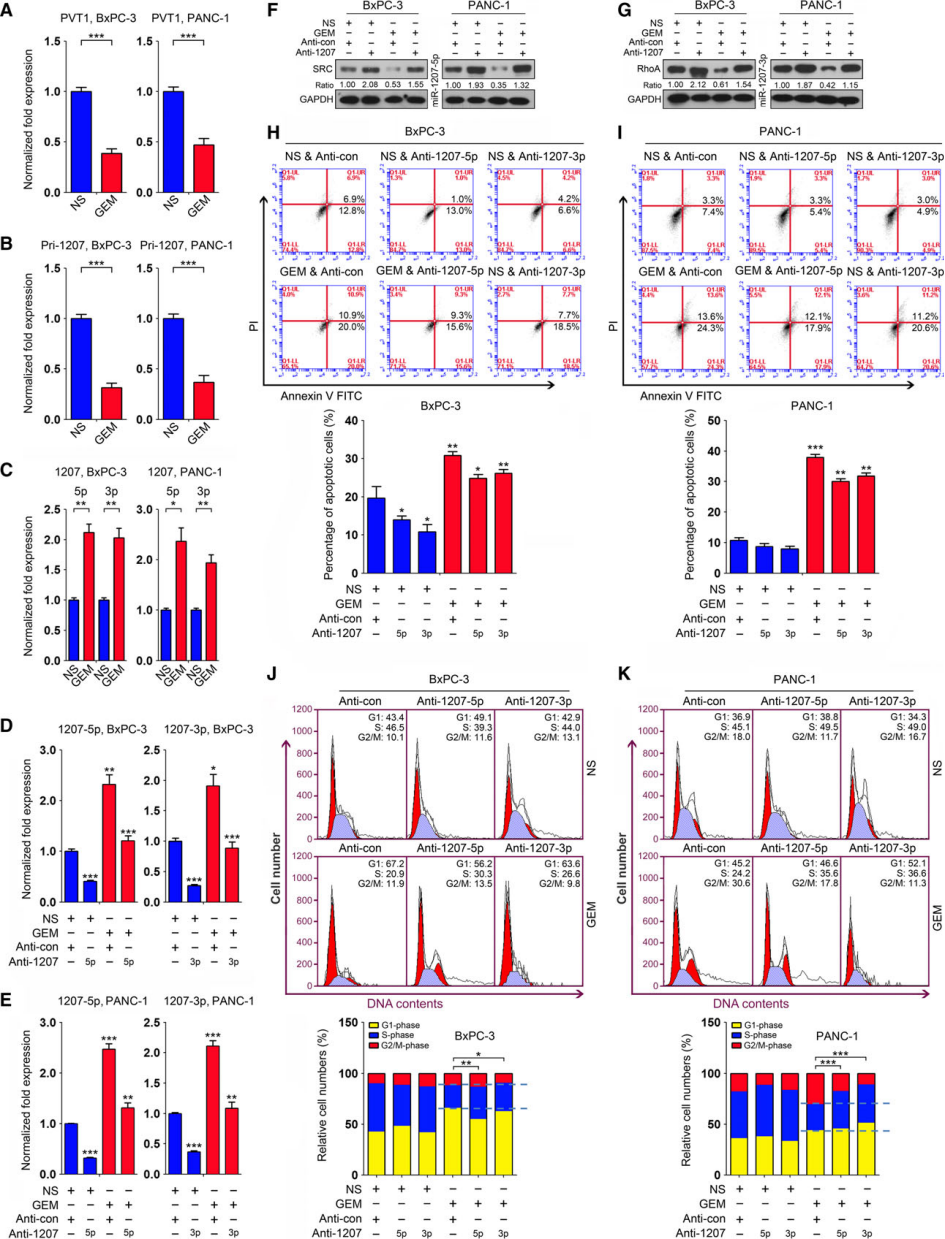
Gemcitabine suppresses PC cell growth by regulating miR‐1207‐induced signaling. (A,B) Expression of PVT1 (A) and primary miR‐1207 (Pri‐1207) (B) was evaluated by qRT‐PCR analysis in BxPC‐3 and PANC‐1 cells treated with gemcitabine. GAPDH served as a loading control. (C) Expression of miR‐1207‐5p and miR‐1207‐3p was assessed in BxPC‐3 and PANC‐1 cells with gemcitabine treatment. U6 snRNA was used as a loading control. (D,E) Expression of miR‐1207 pair was evaluated in BxPC‐3 (D) and PANC‐1 (E) cells in which rescue assays were conducted. (F,G) The protein levels of SRC (F) and RhoA (G) were assessed in BxPC‐3 and PANC‐1 cells in which rescue assays were conducted. (H,I) Apoptosis assays were used in the rescue experiments that were conducted in BxPC‐3 (H) and PANC‐1 (I) cells. The percentage of apoptotic cells is shown (bottom). (J,K) Cell cycle analyses were performed in the rescue experiments that were conducted in BxPC‐3 (J) and PANC‐1 (K) cells. The relative cell numbers at the G1‐, S‐ and G2/M‐phase are shown (bottom ). *P <* *0.05, **P <* *0.01, ***P <* *0.001. Mean ± SD values were calculated from triplicate samples. *P* values were based on Student's *t* test unless otherwise indicated.

We next explored the expression of PVT1 and the miR‐1207 pair in PC cells with gemcitabine treatment. qRT‐PCR analysis revealed that PVT1 expression was downregulated in several PC cell lines with gemcitabine treatment (Figs 4A and S6A). Additionally, we observed that gemcitabine treatment resulted in decreased pri‐miR‐1207 expression and increased the mature miR‐1207 pair levels in these cells (Figs 4B,C and S6B,C). These data suggest that gemcitabine triggers the processing of PVT1 into miR‐1207‐5p/3p. Furthermore, we performed a rescue assay to investigate the role of the miR‐1207 pair in BxPC‐3 and PANC‐1 cells treated with gemcitabine. Accordingly, miR‐1207 inhibitors (anti‐1207‐5p/3p) were transfected into these two cells with or without gemcitabine treatment. qRT‐PCR analysis revealed that gemcitabine treatment increased miR‐1207‐5p or miR‐1207‐3p expression, and this upregulation was dampened by anti‐1207‐5p and anti‐1207‐3p in BxPC‐3 (Fig. 4D) and PANC‐1 (Fig. 4E) cells. We also observed that the SRC (Fig. 4F) or RhoA (Fig. 4G) protein levels were reduced upon gemcitabine treatment and increased with anti‐1207 transfection in BxPC‐3 and PANC‐1 cells, as shown by immunoblots. Subsequent apoptosis and cell cycle analyses indicated that inhibition of the miR‐1207 pair to prevent its induction by gemcitabine treatment led to increased PC cell growth (Figs 4H–K and S7A,B).

Altogether, these findings suggest that miR‐1207 pair‐induced signaling is involved in regulating gemcitabine chemosensitivity in PC cells.

### 3.7 Gemcitabine promotes PVT1 processing by regulating the expression of Drosha and DGCR8 in PC cells

**Figure 5:**
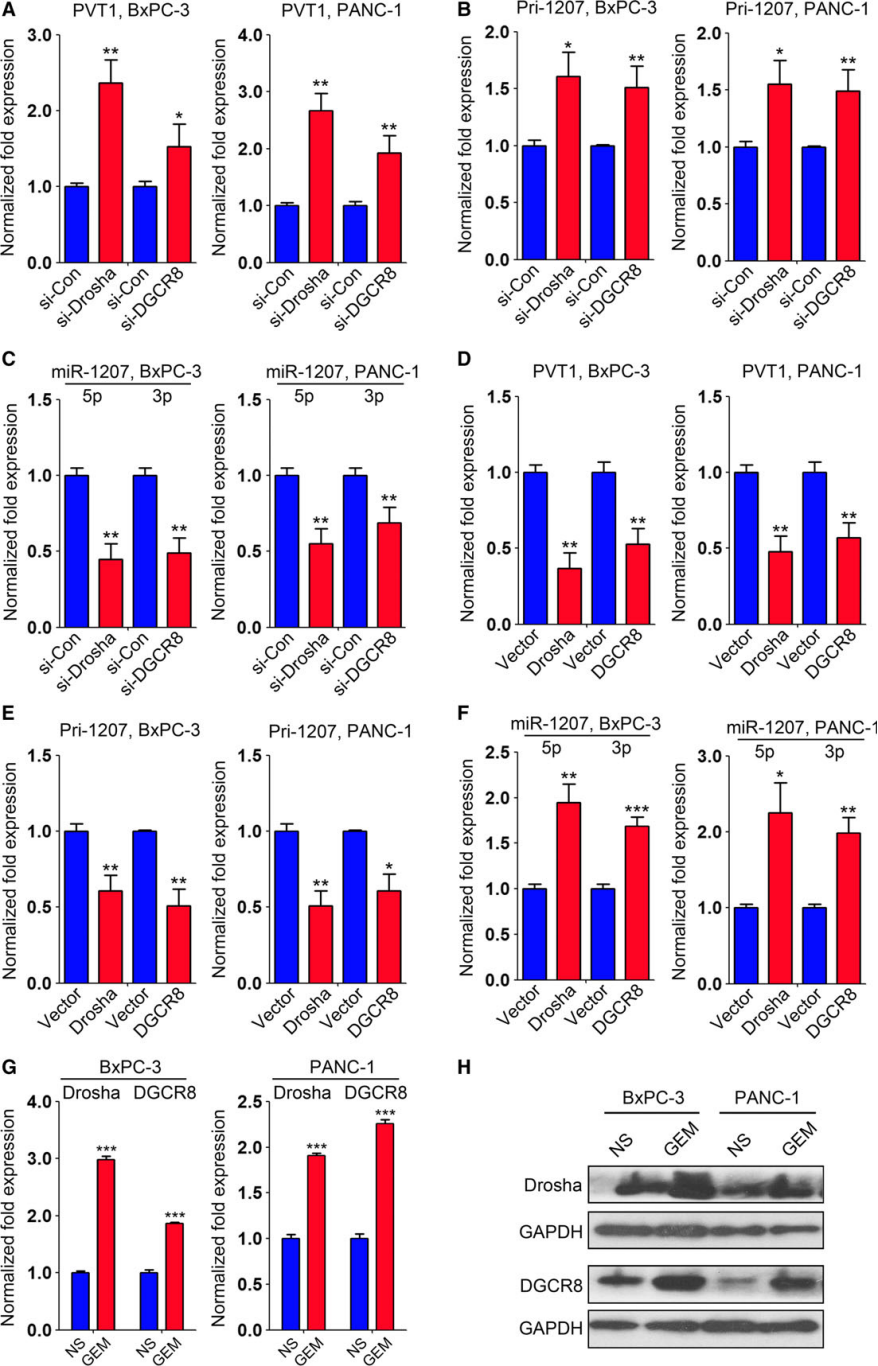
Gemcitabine induces PVT1 processing by triggering the expression of Drosha and DGCR8. (A,B) Expression of PVT1 (A) and Pri‐1207 (B) was evaluated by qRT‐PCR analysis in BxPC‐3 and PANC‐1 cells with inhibition of Drosha and DGCR8. GAPDH was used as a loading control, and data were normalized to si‐Con‐treated cells. (C) Expression of miR‐1207‐5p/3p was assessed in BxPC‐3 and PANC‐1 cells transfected with si‐Drosha or si‐DGCR8. U6 snRNA was used as a loading control, and the data were normalized to si‐Con‐treated cells. (D,E) Expression of PVT1 (D) and Pri‐1207 (E) was evaluated by qRT‐PCR analysis in BxPC‐3 and PANC‐1 cells with overexpression of Drosha and DGCR8. GAPDH was used as a loading control, and the data were normalized to vector‐transfected cells. (F) Expression of miR‐1207‐5p/3p was determined in BxPC‐3 and PANC‐1 cells with overexpression of Drosha or DGCR8. U6 snRNA was used as a loading control, and the data were normalized to vector‐transfected cells. (G,H) The mRNA (G) and protein (H) levels of Drosha and DGCR8 were evaluated in BxPC‐3 and PANC‐1 cells treated with gemcitabine. GAPDH served as a loading control, and the data were normalized to NS‐treated cells. *P <* *0.05, **P <* *0.01, ***P <* *0.001. Mean ± SD values were calculated from triplicate samples. *P* values were based on Student's *t* test unless otherwise indicated.

Our above findings indicated that gemcitabine induced the processing of PVT1 into mature miRNAs in PC cells. To further test this idea, we first considered whether PVT1 processing depended on Drosha/DGCR8. Specific siRNAs for Drosha (si‐Drosha) and DGCR8 (si‐DGCR8) were transfected into BxPC‐3 and PANC‐1 cells (Fig. S8). qRT‐PCR analysis showed that inhibition of Drosha or DGCR8 led to increased PVT1 and pri‐miR‐1207 levels (Fig. 5A,B) and decreased miR‐1207 pair expression (Fig. 5C). Next, we overexpressed Drosha and DGCR8 in these two cell lines (Fig. S9) and evaluated the expression of PVT1, pri‐miR‐1207 and the miR‐1207 pair. qRT‐PCR analysis indicated that PVT1 and pri‐miR‐1207 were downregulated (Fig. 5D,E), whereas the expression of miR‐1207‐5p and miR‐1207‐3p was upregulated (Fig. 5F). These findings suggest that PVT1 processing is Drosha/DGCR8‐dependent in BxPC‐3 and PANC‐1 cells. We further determined the expression of Drosha and DGCR8 in these two cells with gemcitabine treatment using qRT‐PCR analysis and observed that the expression of Drosha and DGCR8 was significantly upregulated (Fig. 5G). Similar results were observed as indicated by immunoblotting analysis (Fig. 5H).

Taken together, our data demonstrate that gemcitabine regulates PVT1 processing via triggering the expression of Drosha and DGCR8.

### 3.8 Inhibition of Drosha and DGCR8 dampens gemcitabine efficacy in PC cells

**Figure 6:**
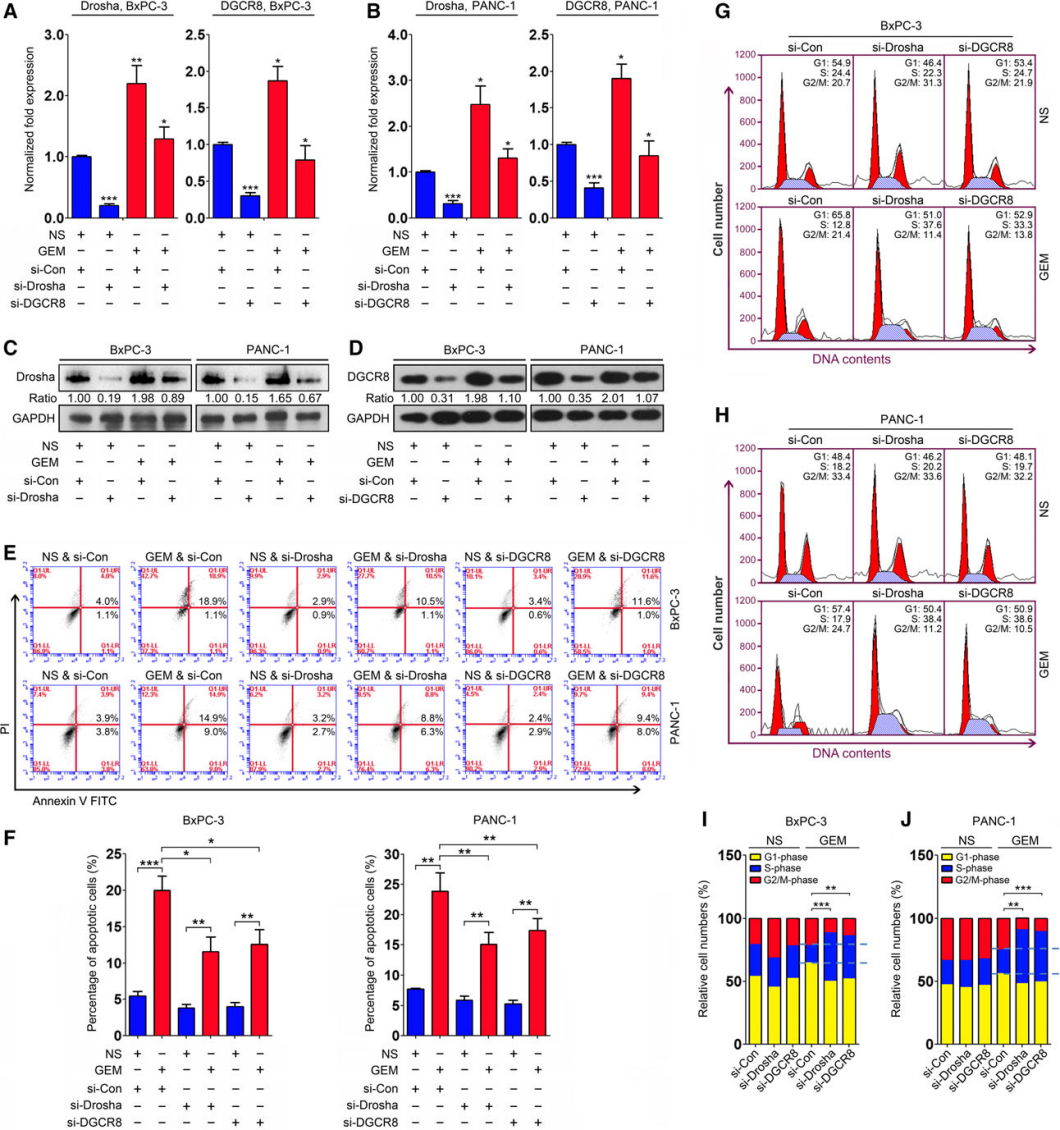
Inhibition of Drosha and DGCR8 reduces the efficacy of gemcitabine in PC cells. (A,B) Expression of Drosha and DGCR8 was evaluated by qRT‐PCR analysis in BxPC‐3 (A) and PANC‐1 (B) cells transfected with specific siRNAs upon gemcitabine treatment. GAPDH served as a loading control, and the data were normalized to cells treated with NS and si‐Con. (C,D) Expression of Drosha (C) and DGCR8 (D) was determined by immunoblotting analyses in BxPC‐3 and PANC‐1 cells transfected with specific siRNAs upon gemcitabine treatment. GAPDH served as a loading control, and the data were normalized to cells treated with NS and si‐Con, and the numbers shown below represent the normalized protein expression levels. (E,F) Apoptosis assays were employed in the rescue experiments that were conducted in BxPC‐3 and PANC‐1 cells (E). The percentage of apoptotic cells is shown in (F). (G–J) Cell cycle analyses were performed in the rescue experiments that were conducted in BxPC‐3 (G) and PANC‐1 (H) cells. The relative cell numbers at G1‐, S‐ and G2/M‐phase are shown in (I) and (J). *P <* *0.05, **P <* *0.01, ***P <* *0.001. Mean ± SD values were calculated from triplicate samples. *P* values were based on Student's *t* test unless otherwise indicated.

As stated above, gemcitabine suppressed PC cell growth by regulating the processing of PVT1 into mature miRNAs by triggering Drosha and DGCR8 expression. We further confirmed these findings via a series of rescue assays. si‐Drosha and si‐DGCR8 were transfected into BxPC‐3 and PANC‐1 cells upon gemcitabine treatment. Expression of Drosha and DGCR8 was determined, which indicated that the gemcitabine‐induced upregulation of Drosha and DGCR8 was suppressed by specific siRNAs for these two genes, as shown by qRT‐PCR and immunoblotting analyses (Fig. 6A–D). Subsequent apoptosis assays indicated that inhibition of Drosha and DGCR8 to prevent its induction by gemcitabine treatment led to decreased programmed cell death in BxPC‐3 and PANC‐1 cells (Fig. 6E,F). We also performed cell cycle analyses in these two cells with the same treatment and observed that Drosha and DGCR8 inhibition to prevent the induction of gemcitabine treatment led to increased cell numbers in S‐phase in BxPC‐3 (Figs 6G,I and S10A) and PANC‐1 (Figs 6H,J and S10B) cells.

Altogether, our data suggest a mechanism in which the efficacy of gemcitabine therapy in PC is mediated via the promotion of PVT1 switch to mature miRNAs.

### 3.9 Gemcitabine‐induced downregulation of Drosha and DGCR8 leads to chemoresistance in AsPC‐1 cells

**Figure 7:**
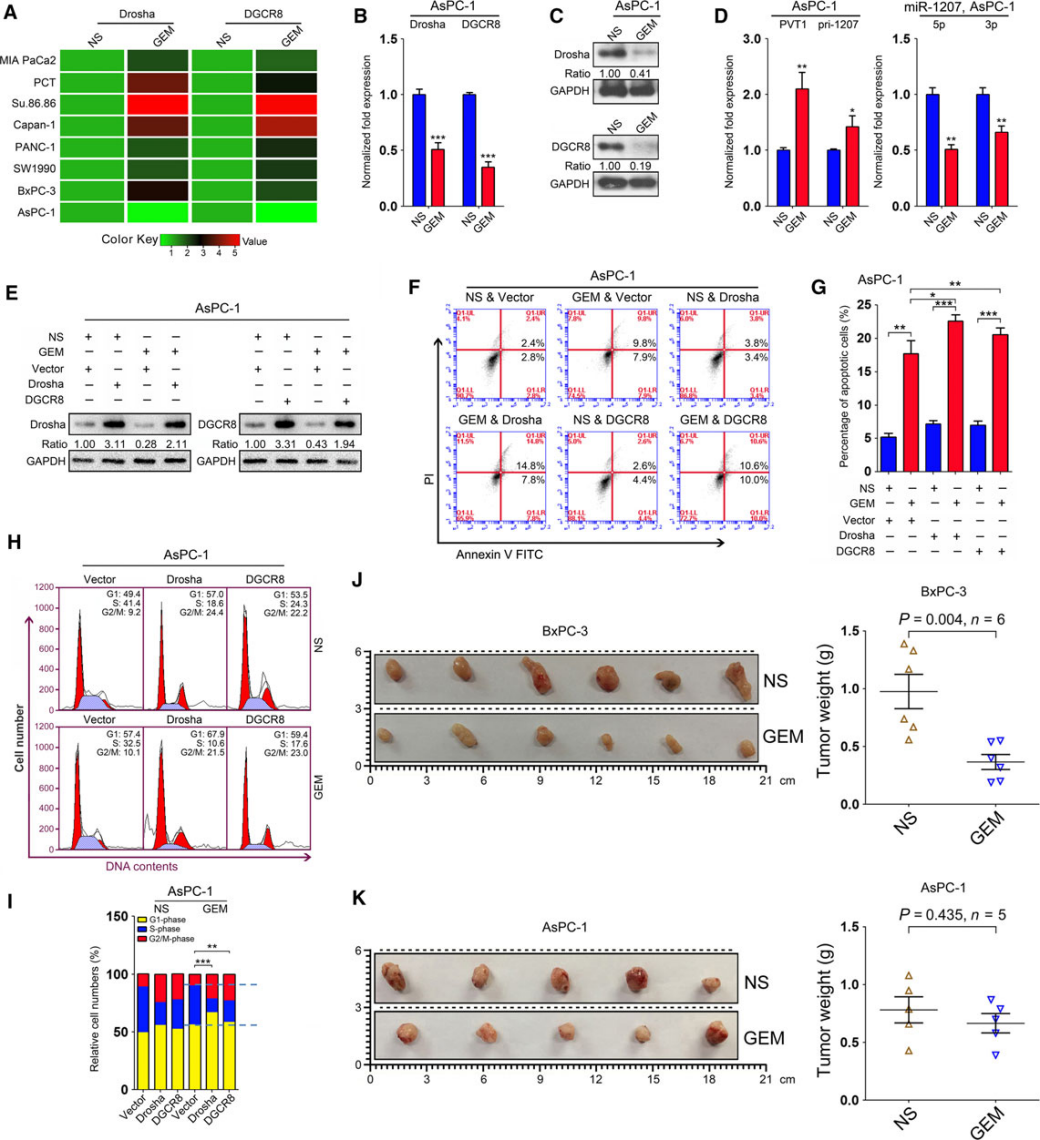
Gemcitabine‐induced downregulation of Drosha and DGCR8 leads to chemoresistance in AsPC‐1 cells. (A) Expression of Drosha and DGCR8 was evaluated by qRT‐PCR analysis in several PC cells with gemcitabine treatment. GAPDH served as a loading control, and the data were normalized to cells treated with NS. (B) Expression of Drosha and DGCR8 was decreased in AsPC‐1 cells upon gemcitabine treatment as shown by qRT‐PCR analysis. GAPDH served as a loading control, and the data were normalized to cells treated with NS. (C) Expression of Drosha and DGCR8 was determined by immunoblotting analysis in AsPC‐1 cells treated with gemcitabine. GAPDH served as a loading control, and the data were normalized to cells treated with NS. The numbers shown below represent the normalized protein expression levels. (D) Expression of PVT1 and pri‐miR‐1207 was upregulated in AsPC‐1 cells upon gemcitabine treatment. GAPDH served as a loading control, and the data were normalized to cells treated with NS. (E) Drosha and DGCR8 expression levels were determined in AsPC‐1 cells upon overexpression of these two proteins with gemcitabine treatment. GAPDH served as a loading control, and the data were normalized to cells treated with NS and vector. The numbers shown below represent the normalized protein expression levels. (F,G) Apoptosis assay was employed in AsPC‐1 cells transfected with overexpression constructs for Drosha or DGCR8, with or without gemcitabine treatment (F). The percentage of apoptotic cells is shown in (G). (H,I) Cell cycle analysis was performed in AsPC‐1 cells upon overexpression of Drosha or DGCR8, with or without gemcitabine treatment (H). The relative cell numbers at G1‐, S‐ and G2/M‐phase are shown in (I). Mean ± SD values were calculated from triplicate samples. (J,K) The effect of gemcitabine on PC cell growth was investigated in BxPC‐3‐ and AsPC‐1‐derived xenograft models. Tumor weights were calculated at the end of the experiment. *P <* *0.05, **P <* *0.01, ***P <* *0.001. *P* values were based on Student's *t* test unless otherwise indicated.

Expression analysis of Drosha and DGCR8 was conducted on several PC cell lines, including BxPC‐3 and PANC‐1, showing that these two genes were significantly upregulated upon gemcitabine treatment (Fig. 7A). Interestingly, we observed that gemcitabine reduced the expression of Drosha and DGCR8 in AsPC‐1 cells, as shown by qRT‐PCR and immunoblotting analyses (Fig. 7B,C). qRT‐PCR analysis revealed that the expression of PVT1 and pri‐miR‐1207 was upregulated, whereas the levels of the miR‐1207 pair were downregulated in gemcitabine‐treated AsPC‐1 cells (Fig. 7D). These data suggest that ASPC‐1 cells exhibit chemoresistance to gemcitabine treatment, clarifying the observation that the half maximal inhibitory concentration value of gemcitabine in AsPC‐1 cells is much higher than that in BxPC‐3 and PANC‐1 cells (Fig. S1B). We further investigated whether the chemosensitivity of gemcitabine could be improved by enforced expression of Drosha and DGCR8 in AsPC‐1 cells. Apoptosis analysis was conducted in AsPC‐1 cells treated with gemcitabine or NS and Drosha or DGCR8 overexpression and it was observed that overexpression of Drosha or DGCR8 led to an increase in apoptosis (Fig. 7E–G). Cell cycle analyses indicated that overexpression of Drosha or DGCR8 resulted in a decrease in the numbers of cells in S‐phase in gemcitabine‐treated AsPC‐1 cells (Figs 7H,I and S11). These data demonstrate that overexpression of Drosha and DGCR8 contributes to improved gemcitabine chemosensitivity in AsPC‐1 cells.

To further confirm the above findings, the effect of gemcitabine on tumor cell growth was evaluated on nude mice subcutaneously injected with BxPC‐3 and AsPC‐1 cells, respectively. We observed that the tumor weights were decreased in BxPC‐3 derived xenograft models upon gemcitabine treatment, whereas no significant difference was observed between gemcitabine treatment and the control group of AsPC‐1 derived xenograft models (Fig. 7J,K).

Taken together, our data indicate that gemcitabine has inhibitory efficacy via Drosha/DGCR8‐induced signaling and also that deregulated Drosha/DGCR8 might contribute to gemcitabine chemoresistance in PC cells.

## 4 Discussion

PVT1 represents a non‐protein coding locus that is co‐overexpressed with the *myc* proto‐oncogene, indicating its role in cancer biology (Carramusa *et al*., 2007; L'Abbate *et al*., 2014; Tseng *et al*., 2014). Recent studies have revealed a complicated function for this lncRNA. For example, PVT1 knockdown leads to reduced protein levels of the major extracellular matrix proteins in mesangial cells, suggesting its regulatory function as a lncRNA (Alvarez *et al*., 2013). Although our previous work indicates that gain‐ or loss‐of PVT1 function affects gemcitabine efficacy in PC cells by transfection with full‐length PVT1 in the sense or antisense orientation, the role of the *pvt1* locus remains unclear as a result of its encoded miRNAs (Beck‐Engeser *et al*., 2008; You *et al*., 2011). These miRNAs exhibit critical functions in cell biology, which presents a major challenge with respect to clarifying the function of the *pvt1* locus (Barsotti *et al*., 2012; Johnsson and Morris, 2014). In the present study, we explored the roles of miR‐1204, miR‐1207‐5p/3p and miR‐1208 in gemcitabine chemotherapy and demonstrated that enforced expression of miR‐1204 and the miR‐1207 pair led to enhanced gemcitabine efficacy. Because miR‐1207‐5p/3p had the most significant impact on gemcitabine chemosensitivity, we next utilized a miRNA target prediction program to screen for functional effectors of the miR‐1207 pair in PC cells. Two well‐known oncogenes, SRC and RhoA, were identified as the *bona fide* targets of miR‐1207‐5p and miR‐1207‐3p, respectively. These findings suggest that gemcitabine exhibits its suppressive impact on PC cell growth by regulating miR‐1207 pair‐induced signaling.

**Figure 8:**
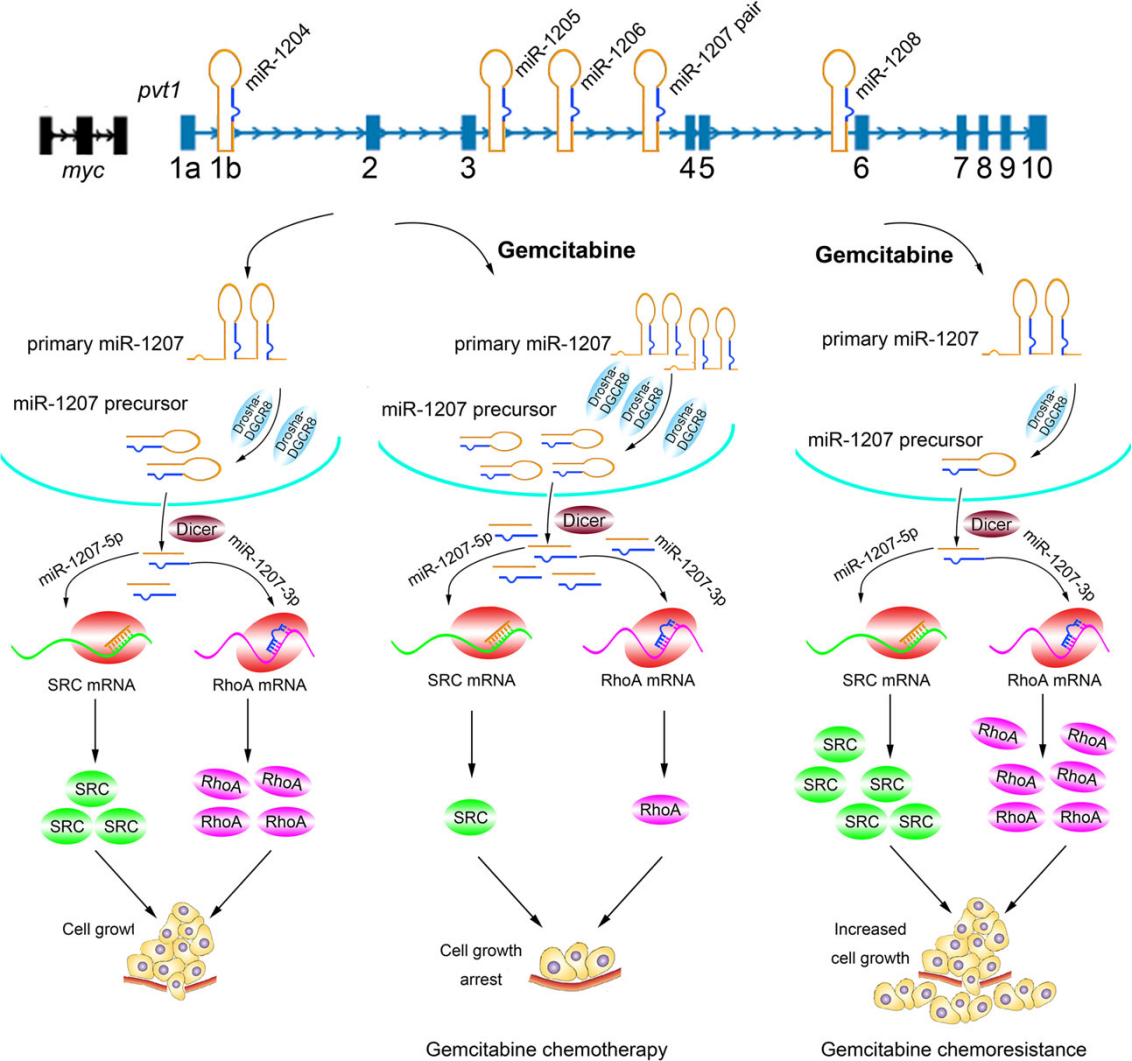
A schematic representation of gemcitabine exhibiting its suppressive effect in PC therapy and the mechanism of gemcitabine chemoresistance in AsPC‐1 cells.

PVT1 levels are downregulated in gemcitabine‐treated BxPC‐3 and PANC‐1 cells, which suggests that this drug may exert its suppressive effect by regulating the expression of this non‐protein coding locus. This regulation might occur at the transcriptional and RNA processing levels. Expression of MYC is downregulated in BxPC‐3 and PANC‐1 cells upon gemcitabine treatment, indicating that decreased PVT1 levels are at least partly attributed to the inhibition of transcription (Fig. S12). Meanwhile, we observed that PVT1 processing is Drosha/DGCR8‐dependent and that gemcitabine alters miRNA processing, as shown by the upregulation of Drosha and DGCR8. These data define a crucial mechanism for gemcitabine treatment in PC via the induction of PVT1 processing into mature miRNAs (Fig. 8). Inhibition of Drosha and DGCR8 leads to a dampened chemosensitivity of gemcitabine, as shown by a rescue assay, which also confirms the above findings. More interestingly, we observed that the expression of Drosha and DGCR8 is significantly downregulated in AsPC‐1 cells upon gemcitabine treatment, indicating a potential mechanism for drug chemoresistance (Fig. 8). Overexpression of Drosha and DGCR8 contributes to an improved chemosensitivity in AsPC‐1 cells by promoting the processing of PVT1 into miRNAs.

## 5 Conclusions

In summary, the findings of the present study define a novel mechanism of gemcitabine chemotherapy and indicate that re‐modification of PVT1 processing and the related signaling may represent a realistic approach for the prevention of PC.

## Author contributions

LY, CL and YZ were responsible for the study concept and design. LY, HW and GY were responsible for the acquisition of data. LY, FZ, JZ, TZ and ZL were responsible for the analysis and interpretation of data. LY, HW and CL were responsible for the drafting of the manuscript. LY, CL, and YZ were responsible for critical revision of the manuscript with respect to important intellectual content. LY, HW, GY and ZL were responsible for the statistical analysis. LY and CL obtained funding. TZ, ZL and ZL were responsible for administrative, technical or material support. CL and YZ were responsible for study supervision.

## Supporting information


**Fig. S1**. The impact of PVT1 inhibition on PC cell growth.

**Fig. S2**. Overexpression of *pvt1*‐encoded miRNAs is determined in PC cell lines.

**Fig. S3**. The impact of *pvt1*‐encoded miRNAs on PC cell growth.

**Fig. S4**. The impact of miR‐1208 on PC cell growth.

**Fig. S5**. Overexpression of miR‐1207 pair leads to decreased cell numbers at S‐phase.

**Fig. S6**. Gemcitabine promotes the processing of PVT1 in PC cell lines.

**Fig. S7**. Inhibition of miR‐1207 pair leads to increased cell numbers at S‐phase.

**Fig. S8**. The expression of Drosha and DGCR8 is determined in PC cells with the inhibition of Drosha and DGCR8.

**Fig. S9.** The expression of Drosha and DGCR8 is determined in PC cells with the overexpression of Drosha and DGCR8.

**Fig. S10**. Inhibition of Drosha or DGCR8 leads to increased cell numbers at S‐phase.

**Fig. S11.** Overexpression of Drosha or DGCR8 leads to decreased cell numbers at S‐phase in AsPC‐1 cells.

**Fig. S12.** The expression of MYC is determined in PC cells with gemcitabine treatment.

**Table S1.** Characterization of 10 pairs of PC patients.
**Table S2.** Sequences of miRNAs and U6 snRNA primers.
**Table S3.** Primers used for the construction of luciferase reporters, as well as qRT‐PCR analyses of mRNAs.
