# Supplementary figures and images for: Gemcitabine exhibits a suppressive effect on pancreatic cancer cell growth by regulating processing of PVT1 to miR1207
Source: Mol Oncol. 2018 Oct 30;12(12):2147–64. doi: 10.1002/1878-0261.12393 (PMC6275279; doi:10.1002/1878-0261.12393)

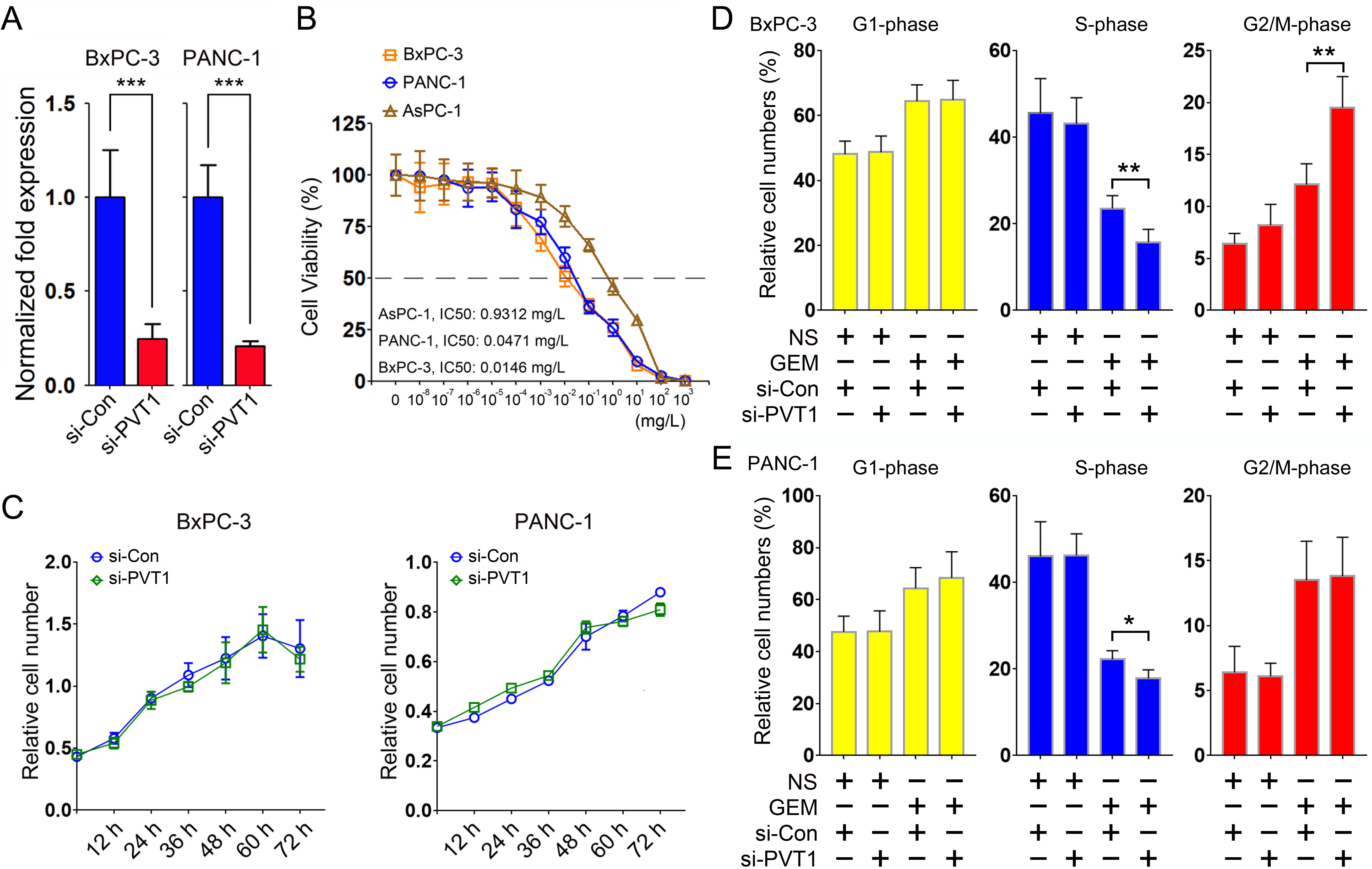

Supplement: Supplementary file 1 — Fig. S1. The impact of PVT1 inhibition on PC cell growth. [file MOL2-12-2147-s001.jpg]

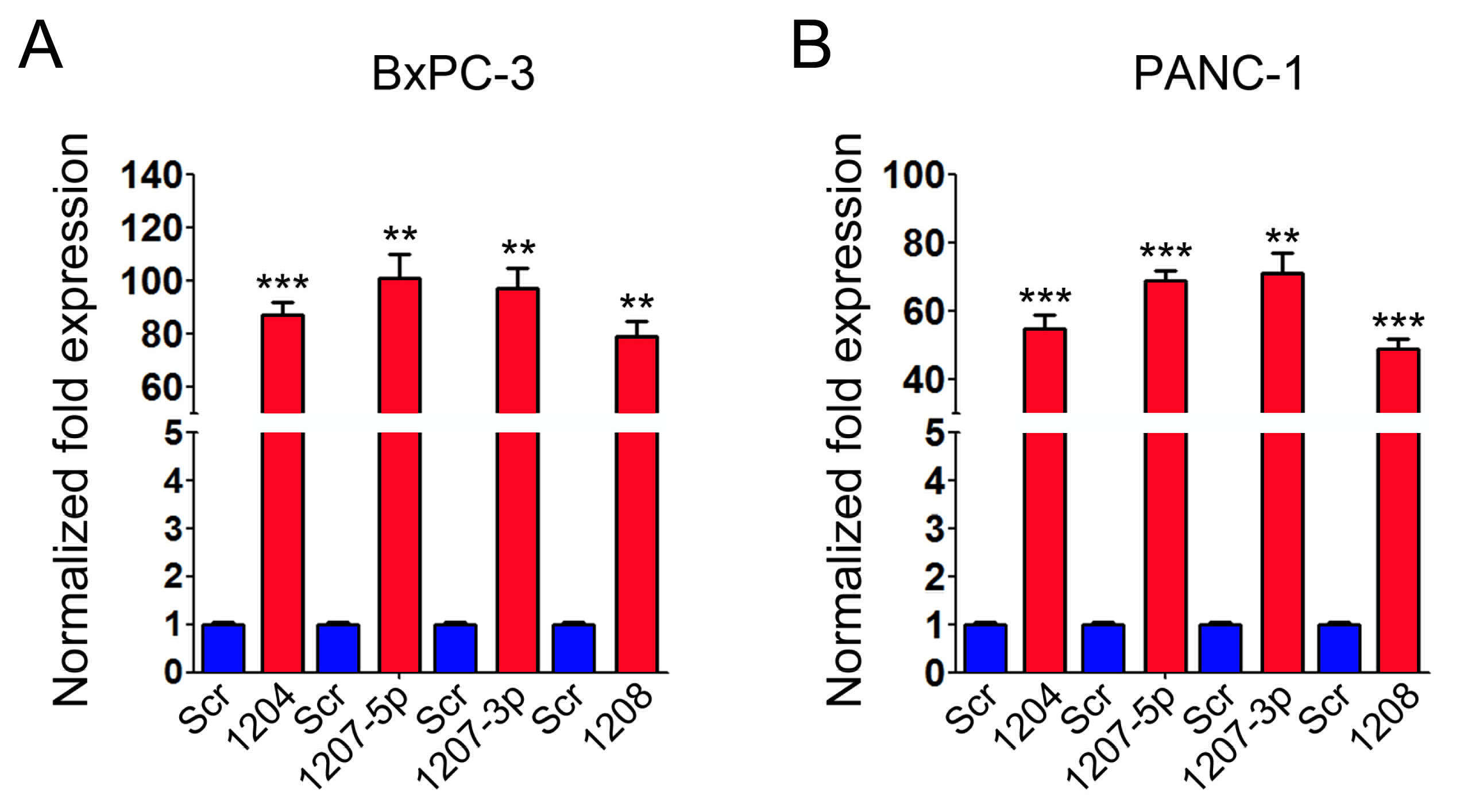

Supplement: Supplementary file 2 — Fig. S2. Overexpression of pvt1‐encoded miRNAs is determined in PC cell lines. [file MOL2-12-2147-s002.jpg]

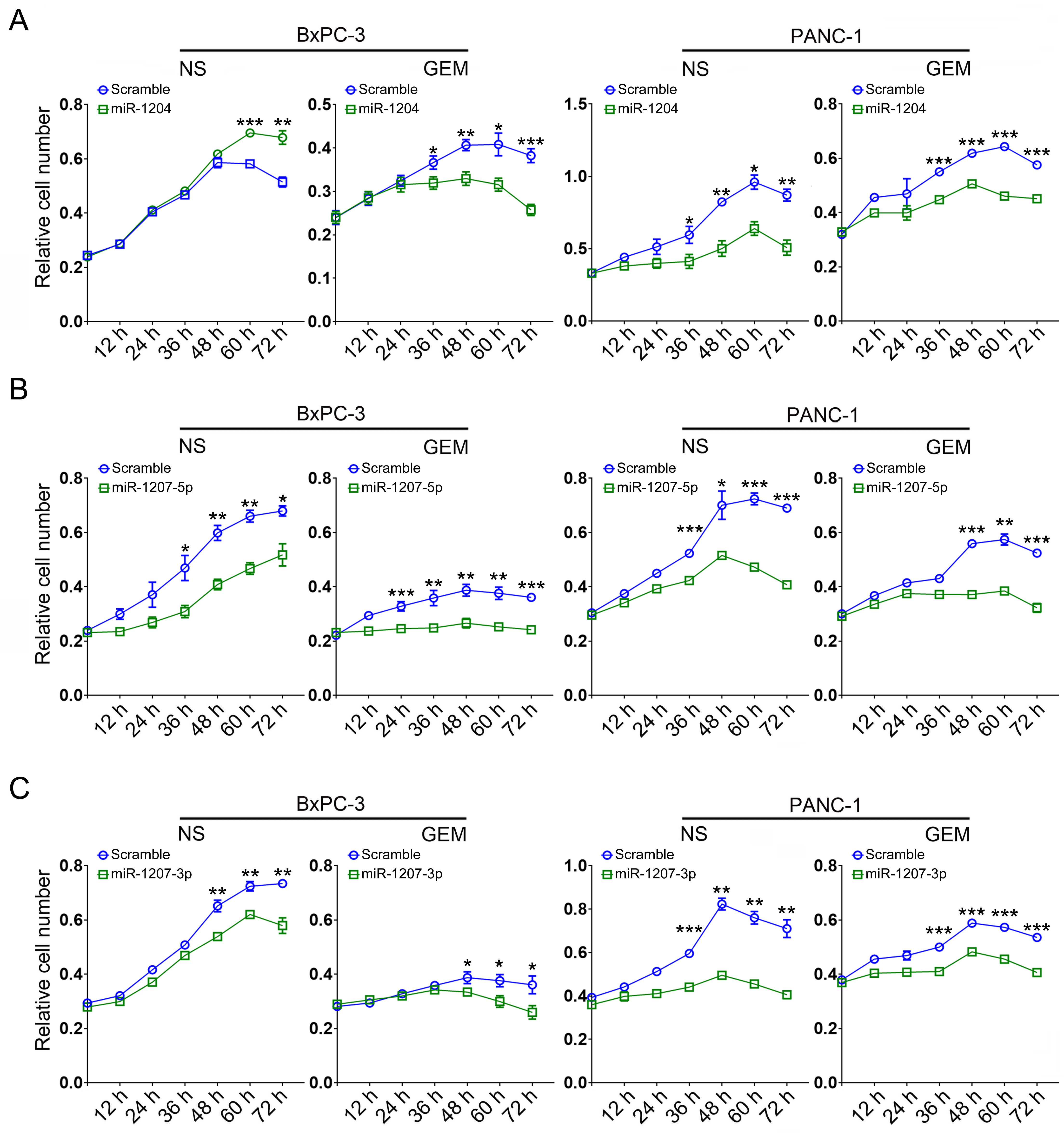

Supplement: Supplementary file 3 — Fig. S3. The impact of pvt1‐encoded miRNAs on PC cell growth. [file MOL2-12-2147-s003.jpg]

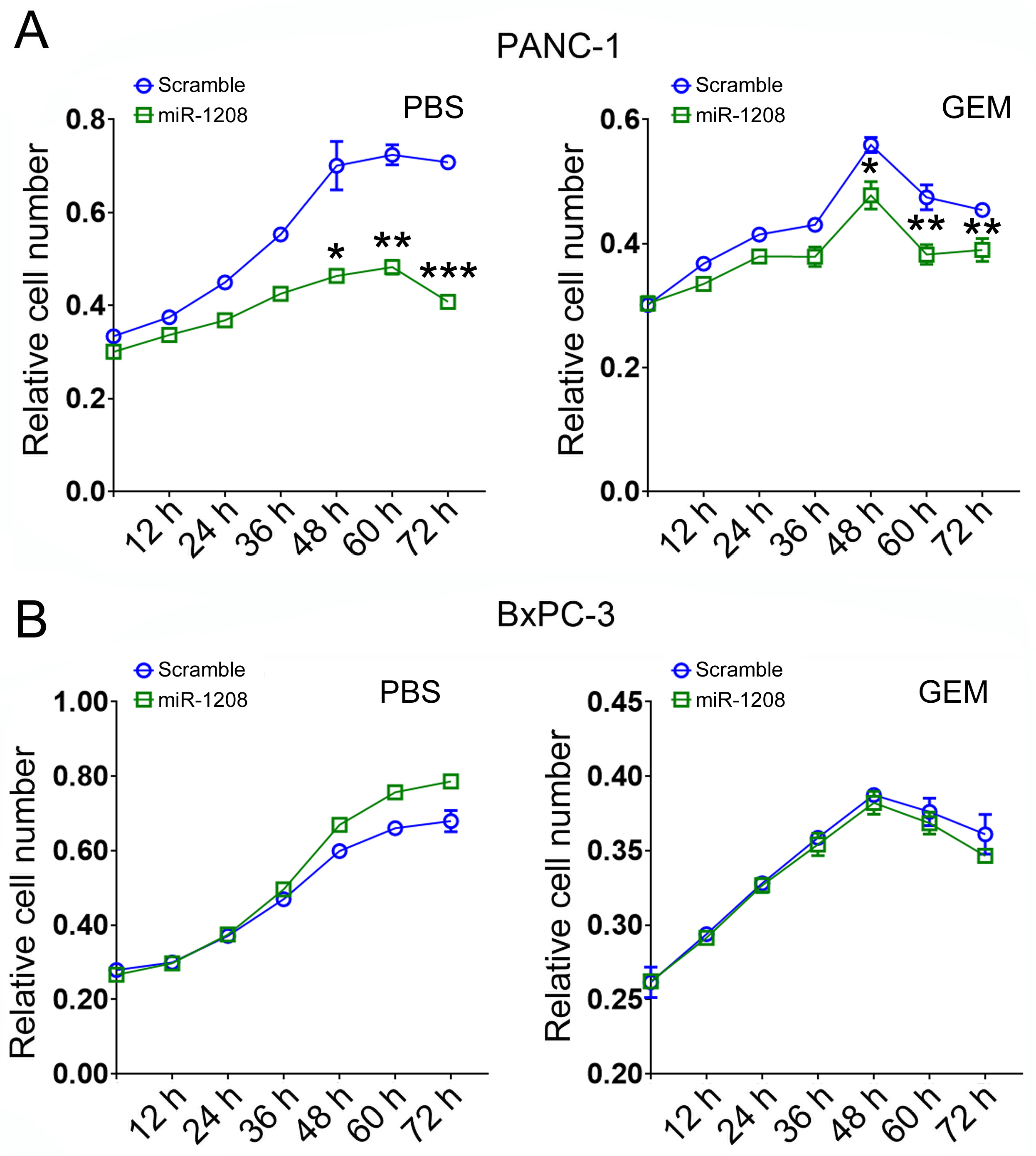

Supplement: Supplementary file 4 — Fig. S4. The impact of miR‐1208 on PC cell growth. [file MOL2-12-2147-s004.jpg]

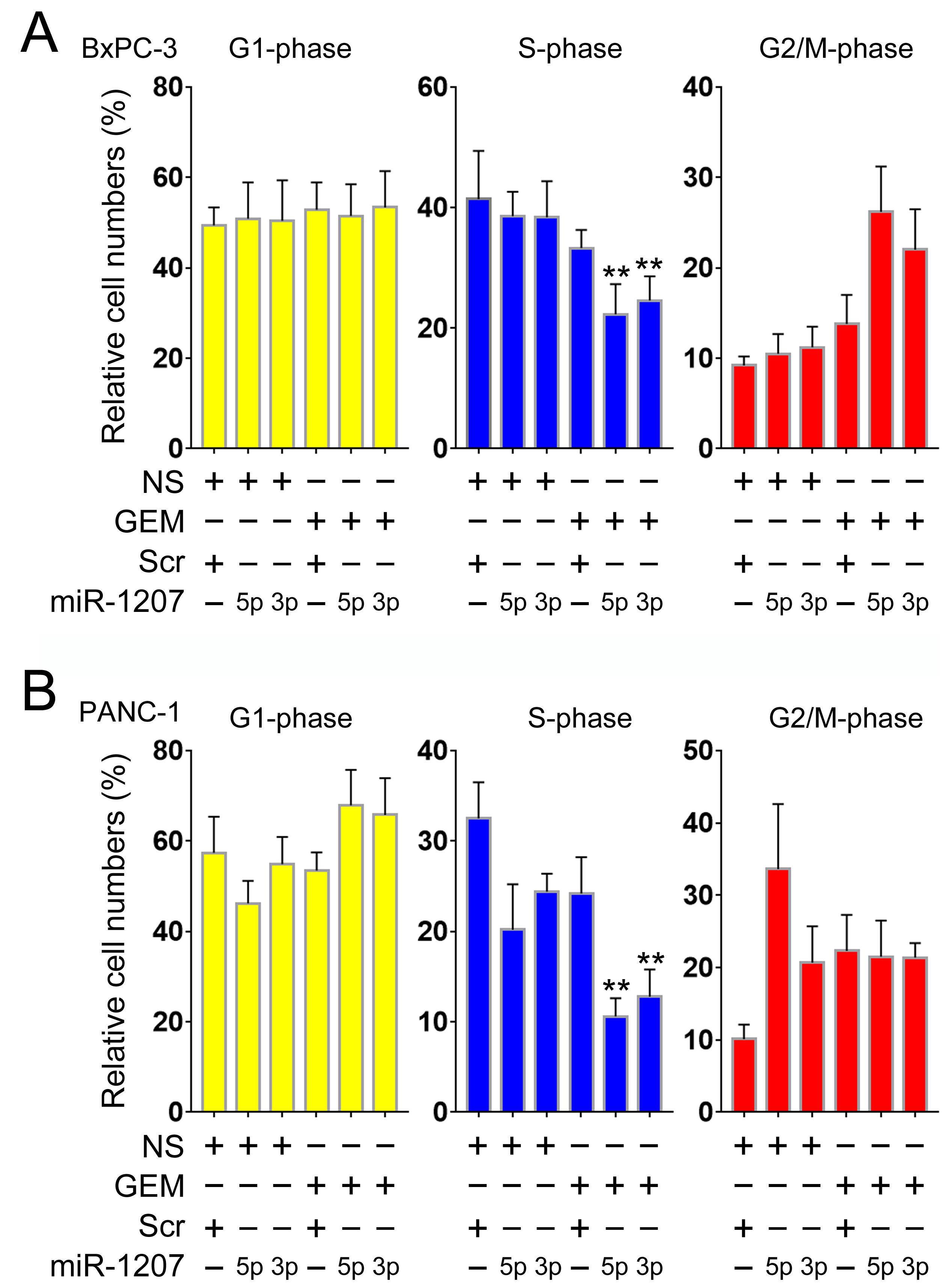

Supplement: Supplementary file 5 — Fig. S5. Overexpression of miR‐1207 pair leads to decreased cell numbers at S‐phase. [file MOL2-12-2147-s005.jpg]

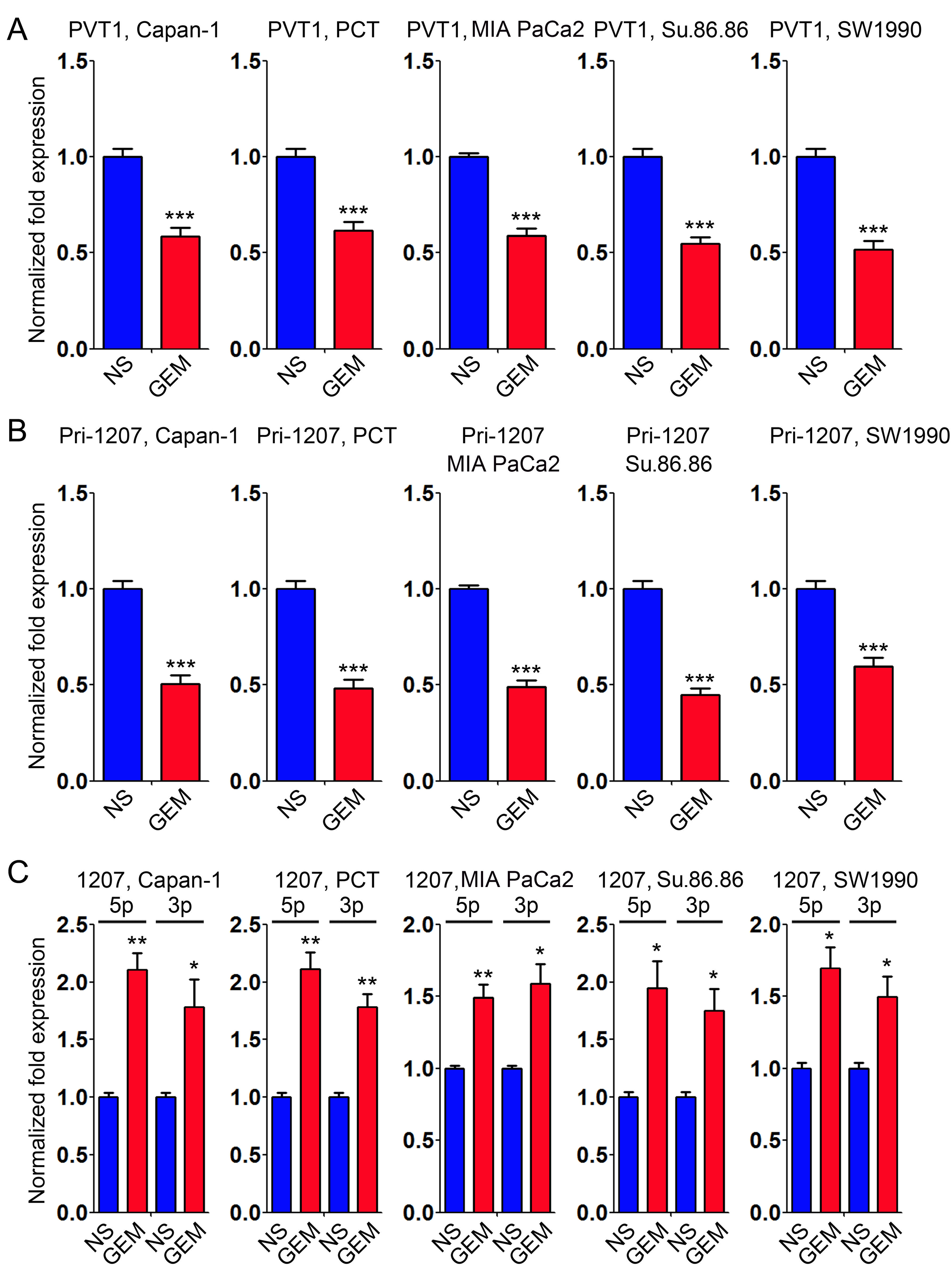

Supplement: Supplementary file 6 — Fig. S6. Gemcitabine promotes the processing of PVT1 in PC cell lines. [file MOL2-12-2147-s006.jpg]

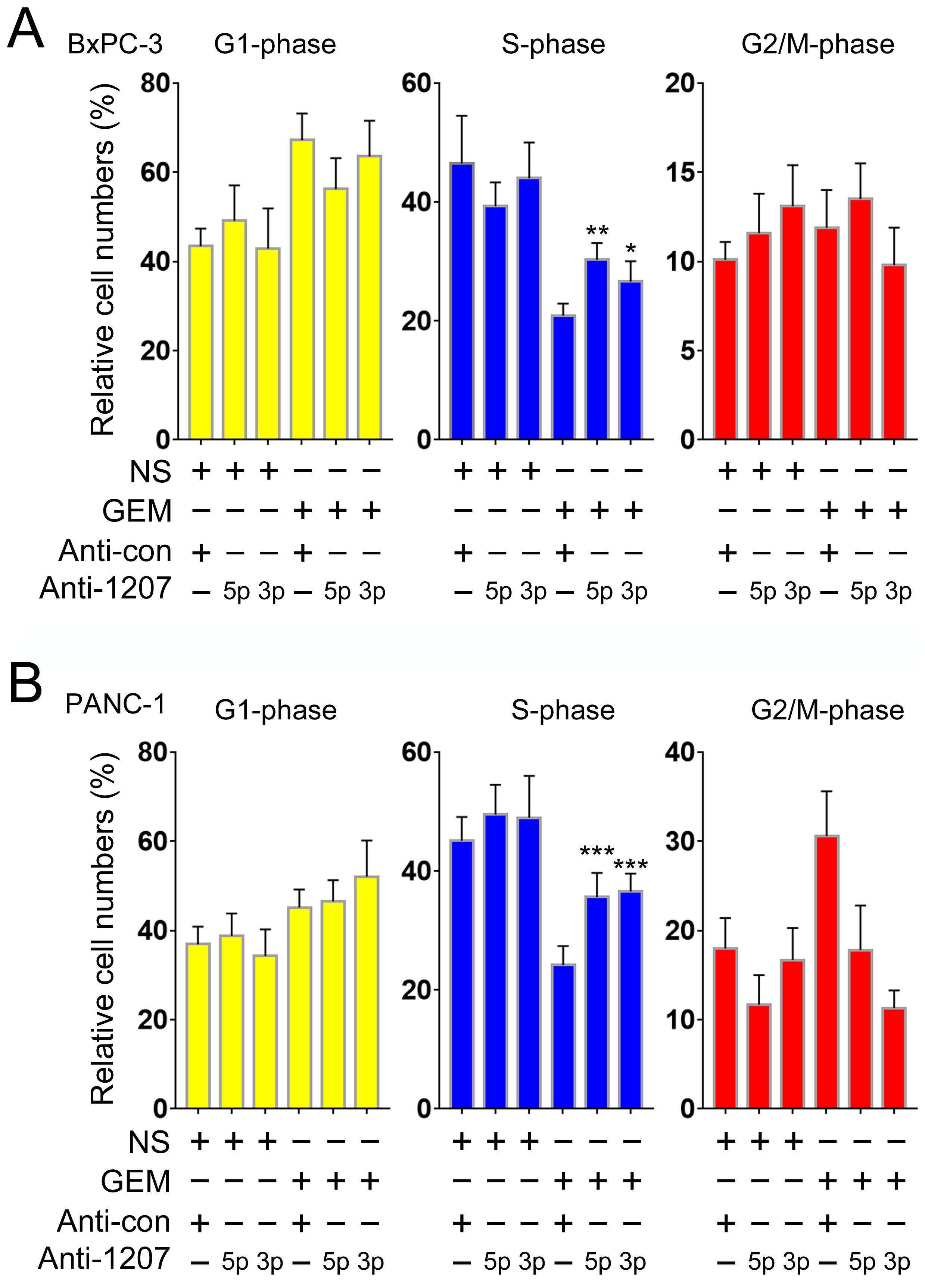

Supplement: Supplementary file 7 — Fig. S7. Inhibition of miR‐1207 pair leads to increased cell numbers at S‐phase. [file MOL2-12-2147-s007.jpg]

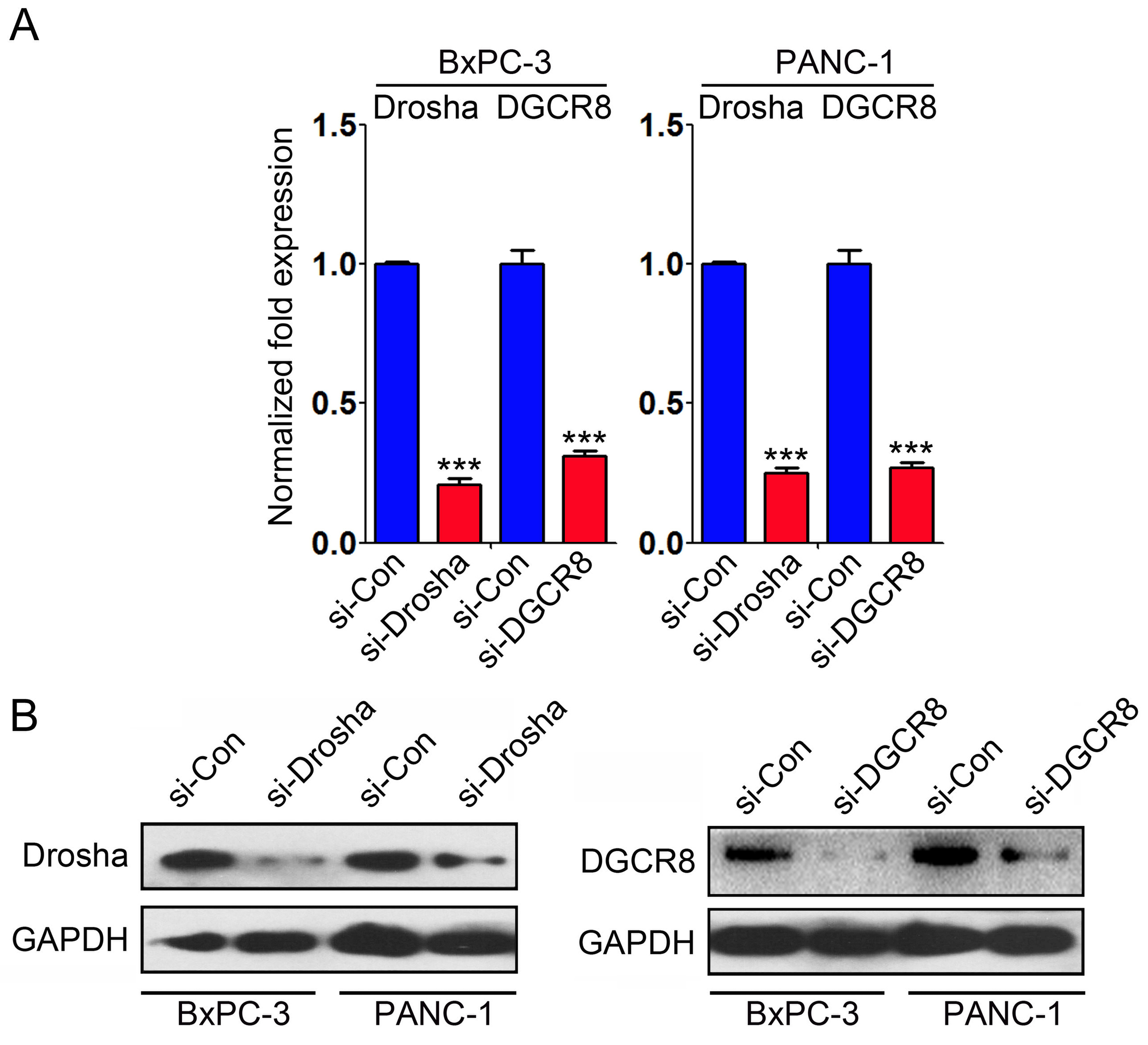

Supplement: Supplementary file 8 — Fig. S8. The expression of Drosha and DGCR8 is determined in PC cells with the inhibition of Drosha and DGCR8. [file MOL2-12-2147-s008.jpg]

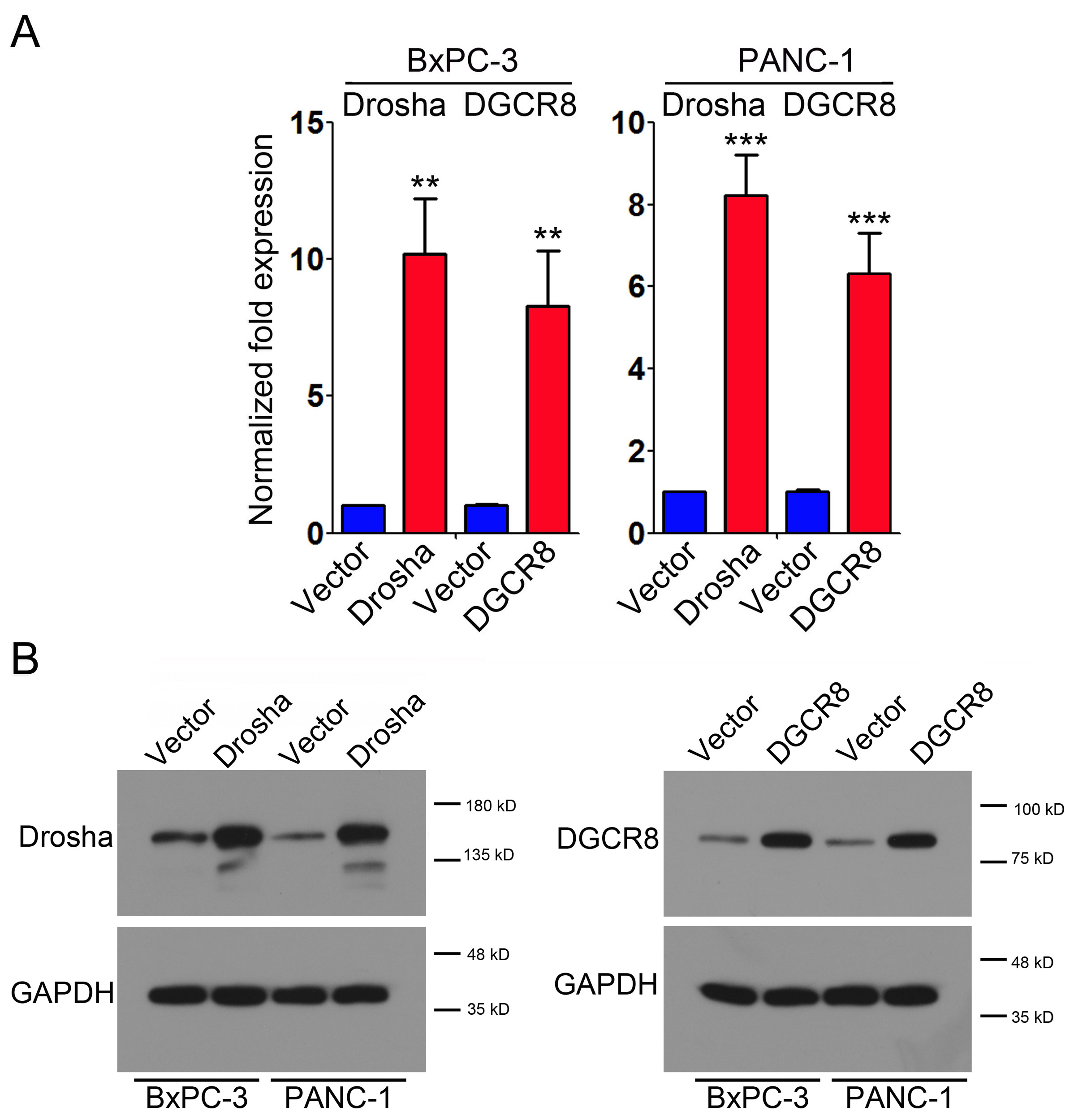

Supplement: Supplementary file 9 — Fig. S9. The expression of Drosha and DGCR8 is determined in PC cells with the overexpression of Drosha and DGCR8. [file MOL2-12-2147-s009.jpg]

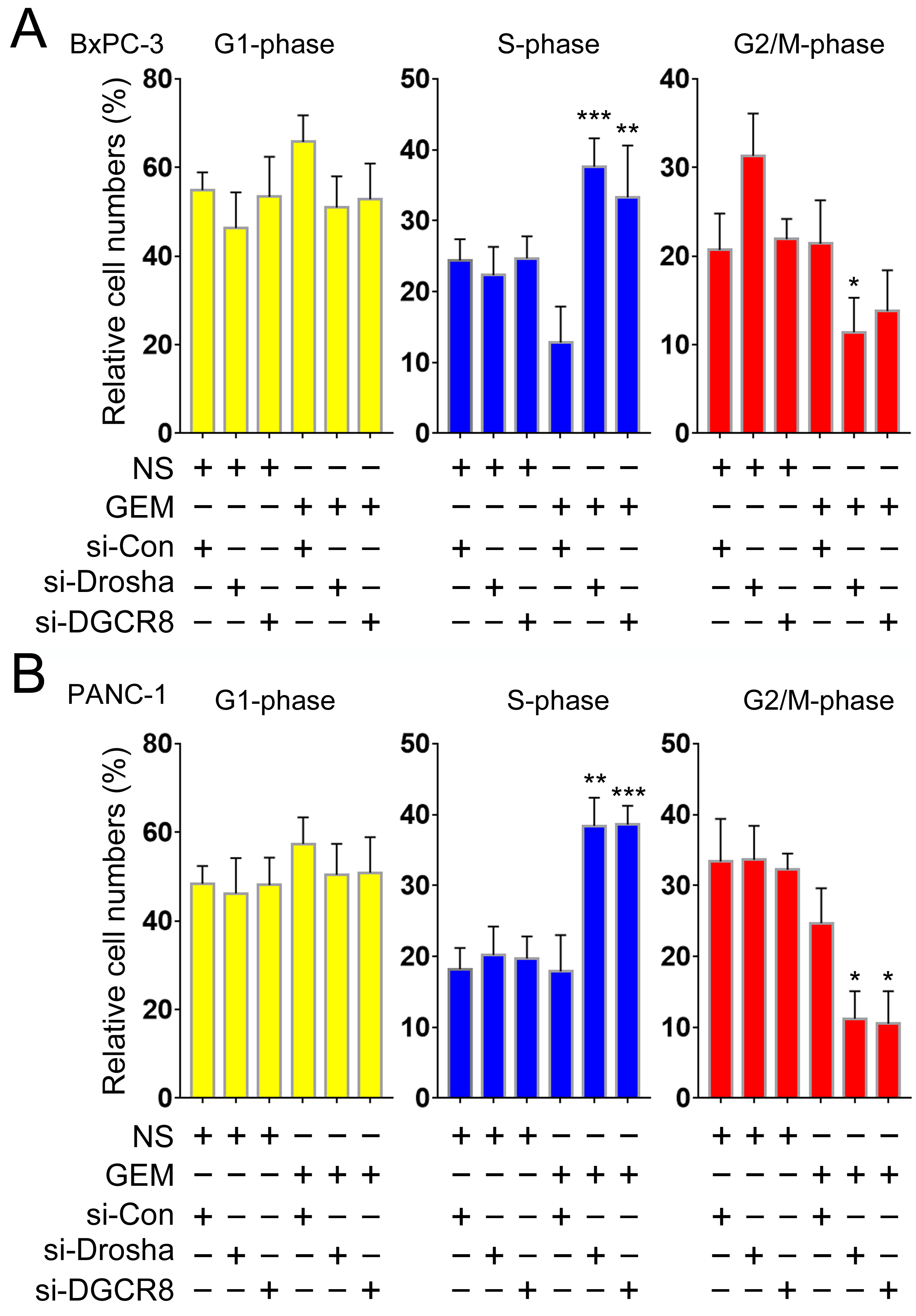

Supplement: Supplementary file 10 — Fig. S10. Inhibition of Drosha or DGCR8 leads to increased cell numbers at S‐phase. [file MOL2-12-2147-s010.jpg]

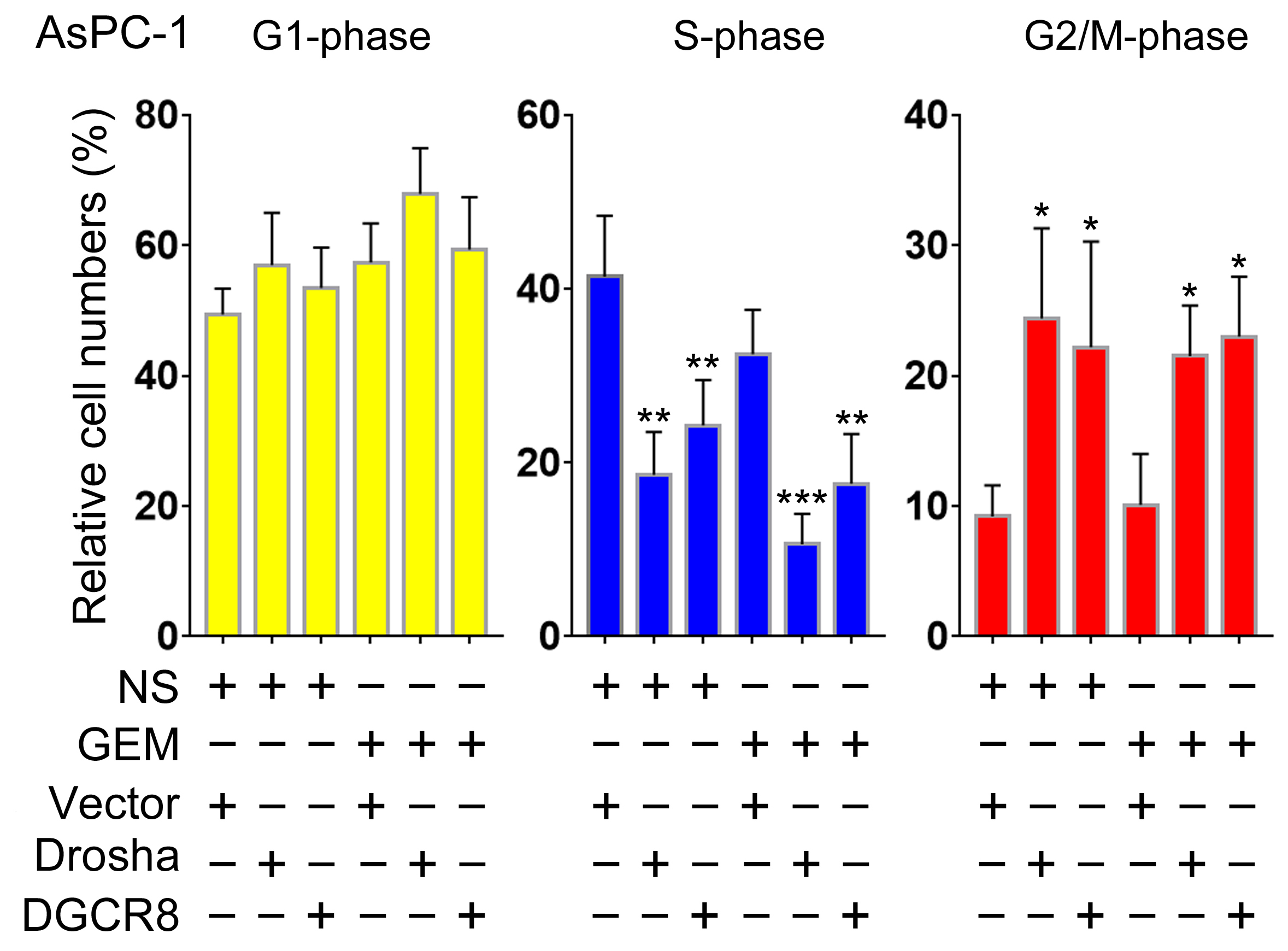

Supplement: Supplementary file 11 — Fig. S11. Overexpression of Drosha or DGCR8 leads to decreased cell numbers at S‐phase in AsPC‐1 cells. [file MOL2-12-2147-s011.jpg]

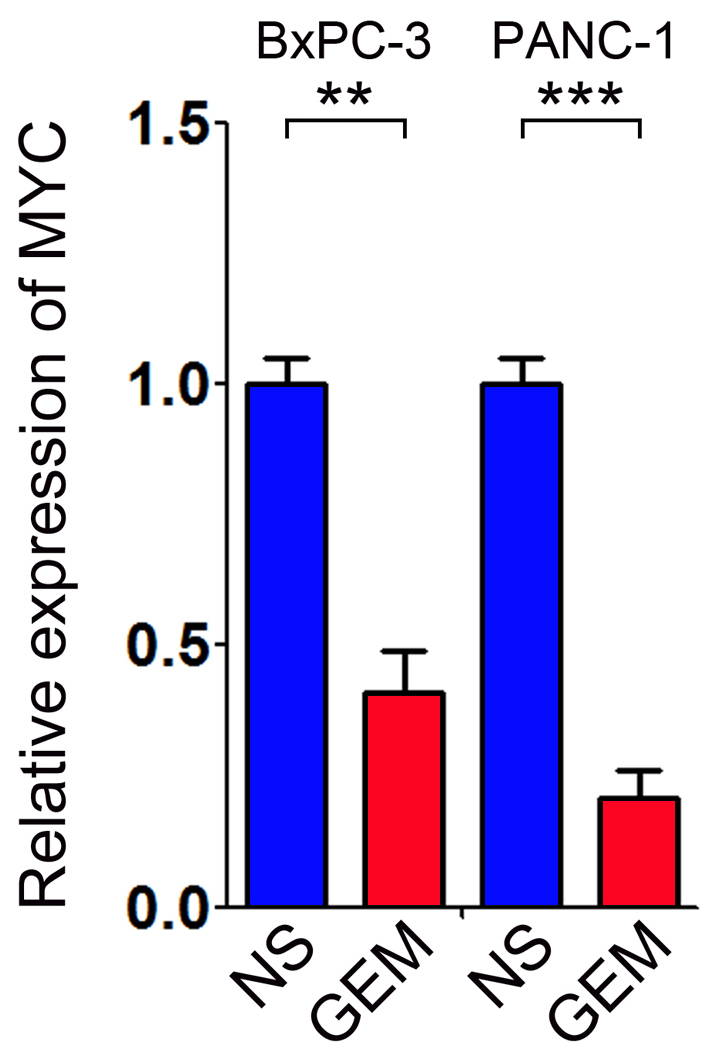

Supplement: Supplementary file 12 — Fig. S12. The expression of MYC is determined in PC cells with gemcitabine treatment. [file MOL2-12-2147-s012.jpg]
