# Supplementary material for: Gemcitabine exhibits a suppressive effect on pancreatic cancer cell growth by regulating processing of PVT1 to miR1207
Source: Mol Oncol. 2018 Oct 30;12(12):2147–64. doi: 10.1002/1878-0261.12393 (PMC6275279; doi:10.1002/1878-0261.12393)
Supplement: Supplementary file 13 — Table S1. Characterization of 10 pairs of PC patients. Table S2. Sequences of miRNAs and U6 snRNA primers. Table S3. Primers used for the construction of luciferase reporters, as well as qRT‐PCR analyses of mRNAs. [file MOL2-12-2147-s013.docx]

**Supplementary Table 1. Characterization of 10 paired of PC patients.**

| No | Age | Gender | Location | Tumor staging | Lymph node staging | TNM staging | Grading |  |  |
| --- | --- | --- | --- | --- | --- | --- | --- | --- | --- |
| 1 | 60 | Male | head | T3 | N1 | ⅡB | G1 |  |  |
| 2 | 46 | Female | head | T2 | N0 | ⅠB | G3 |  |  |
| 3 | 34 | Male | body-tail | T2 | N0 | ⅠB | G1 |  |  |
| 4 | 58 | Male | head | T2 | N0 | ⅠB | G2 |  |  |
| 5 | 65 | Male | body-tail | T3 | N1 | ⅡB | G2 |  |  |
| 6 | 48 | Male | head | T3 | N1 | ⅡB | G2 |  |  |
| 7 | 56 | Female | body-tail | T3 | N1 | ⅡB | G2 |  |  |
| 8 | 58 | Female | body-tail | T3 | N0 | ⅡA | G1 |  |  |
| 9 | 56 | Male | body-tail | T3 | N1 | ⅡB | G1 |  |  |
| 10 | 69 | Female | Body-tail | T2 | N0 | ⅠB | G2 |  |  |
|  | | | | | | | | | |

**Supplementary Table 2. Sequences of miRNAs and U6 snRNA primers.**

| Name | Primer sequence (from 5’→3’) |
| --- | --- |
| Human U6 snRNA  hsa-miR-1204  Pri-miR-1204  Pre-miR-1204  Mature miR-1204  hsa-miR-1205  Pri-miR-1205  Pre-miR-1205  Mature miR-1205  hsa-miR-1206  Pri-miR-1206  Pre-miR-1206  Mature miR-1206  hsa-miR-1207-5p  hsa-miR-1207-3p  Pri-miR-1207  Pre-miR-1207  Mature miR-1207-5p  Mature  miR-1207-3p  hsa-miR-1208  Pri-miR-1208  Pre-miR-1208  Mature miR-1208 | Reverse transcription: CGCTTCACGAATTTGCGTGTCAT  Forward: GCTTCGGCAGCACATATACTAAAAT  Reverse: CGCTTCACGAATTTGCGTGTCAT  TCGTGGCCTGGTCTCCATTAT  Forward: CGCAGATCGACCGTGTTAT  Reverse: CGCAGATCGACCGTGTTAT  Reverse transcription: GTCGTATCCAGTGCAGGGTCCGAGGTATTCGCACTGGATACGACACCACGAGG  Forward: ACCTCGTGGCCTGGTCTCC  Reverse: TCGTGGCCTGGTCTCCATTAT  Probe: TGGATACGACACCACGAG  Reverse transcription: GTCGTATCCAGTGCAGGGTCCGAGGTATTCGCACTGGATACGACATAATG  Forward: GCTCGTGGCCTGGTCTC  Reverse: CAGTGCAGGGTCCGAGGT  TCTGCAGGGTTTGCTTTGAG  Forward: CGCAGATCGACCGTGTTAT  Reverse: CGCAGATCGACCGTGTTAT  Reverse transcription: GTCGTATCCAGTGCAGGGTCCGAGGTATTCGCACTGGATACGACACAGACTCC  Forward: GCCTCTGCAGGGTTTGCTT  Reverse: TCGTGGCCTGGTCTCCATTAT  Probe: TGGATACGACACAGACTCC  Reverse transcription: GTCGTATCCAGTGCAGGGTCCGAGGTATTCGCACTGGATACGACCTCAAA  Forward: GCCTCTGCAGGGTTTGC  Reverse: CAGTGCAGGGTCCGAGGT  TGTTCATGTAGATGTTTAAGC  Forward: CGCAGATCGACCGTGTTAT  Reverse: CGCAGATCGACCGTGTTAT  Reverse transcription: GTCGTATCCAGTGCAGGGTCCGAGGTATTCGCACTGGATACGACCAGCGTTCAC  Forward: CGCAGATCGACCGTGTTAT  Reverse: TCGTGGCCTGGTCTCCATTAT  Probe: TGGATACGACCAGCGTTCAC  Reverse transcription: GTCGTATCCAGTGCAGGGTCCGAGGTATTCGCACTGGATACGAC GCTTAA  Forward: GCGCGTGTTCATGTAGATGT  Reverse: CAGTGCAGGGTCCGAGGT  TGGCAGGGAGGCTGGGAGGGG  TCAGCTGGCCCTCATTTC  Forward: CGCAGATCGACCGTGTTAT  Reverse: GTGCAGGGTCCGAGGT  Reverse transcription: GTCGTATCCAGTGCAGGGTCCGAGGTATTCGCACTGGATACGACACAGAAGTGCTG  Forward: GGCTGGCAGGGAGGCTG  Reverse: TCGTGGCCTGGTCTCCATTAT  Probe: ATACGACACAGAAGTGCTG  Reverse transcription: GTCGTATCCAGTGCAGGGTCCGAGGTATTCGCACTGGATACGACCCCCTC  Forward: TGGCAGGGAGGCTGG  Reverse: CAGTGCAGGGTCCGAGGT  Reverse transcription: GTCGTATCCAGTGCAGGGTCCGAGGTATTCGCACTGGATACGACGAAATG  Forward: GCGTCAGCTGGCCCT  Reverse: CAGTGCAGGGTCCGAGGT  TCACTGTTCAGACAGGCGGA  Forward: CGCGCAATAATACATGGTTG  Reverse: GTGCAGGGTCCGAGGT  Reverse transcription: GTCGTATCCAGTGCAGGGTCCGAGGTATTCGCACTGGATACGACCCACCACAG  Forward: GCAGAATCACTGTTCAGACAGG  Reverse: TCGTGGCCTGGTCTCCATTAT  Probe: TGGATACGACCCACCACAG  Reverse transcription: GTCGTATCCAGTGCAGGGTCCGAGGTATTCGCACTGGATACGACTCCGCC  Forward: GCGCCTCACTGTTCAGACA  Reverse: CAGTGCAGGGTCCGAGGT |

**Supplementary Table 3. Primers used for the construction of luciferase reporters, qRT-PCR analyses of mRNAs.**

| Primer name | Primer sequence (from 5’→3’) | Used for |
| --- | --- | --- |
| pRL-TK-SRC-3’UTR  RL-TK-RhoA-3’UTR  PVT1-qPCR  MYC-qPCR  Drosha-qPCR  DGCR8-qPCR  GAPDH-qPCR | Forward: TCTAGAAAAGCCCAAGTTC  Reverse: GCGGCCGCCTTTATTCTATT  Forward: TCTAGAAAAGCCCAAGTTC  Reverse: GCGGCCGCCTTTATTCTATT  Forward: CGACGACGAGCTGCGAGCAA  Reverse: GCCACGTGGGCGATGAAGTTCGTA  Forward: GGCTCCTGGCAAAAGGTCA  Reverse: CTGCGTAGTTGTGCTGATGT  Forward: TGTCACAGAATGTCGTTCCAC  Reverse: GGGCCTAAAGGATGGTGCT  Forward: GCAGAGGTAATGGACGTTGG  Reverse: AGAGAAGCTCCGTAGAAGTTGAA  Forward: CTGGGCTACACTGAGCACC  Reverse: AAGTGGTCGTTGAGGGCAATG | Plasmid construction  Plasmid construction  qRT-PCR  qRT-PCR  qRT-PCR  qRT-PCR  qRT-PCR |
