# Supplementary material for: Gemcitabine exhibits a suppressive effect on pancreatic cancer cell growth by regulating processing of PVT1 to miR1207
Source: Mol Oncol. 2018 Oct 30;12(12):2147–64. doi: 10.1002/1878-0261.12393 (PMC6275279; doi:10.1002/1878-0261.12393)
Supplement: Supplementary file 14 [file MOL2-12-2147-s014.docx]

**Supplementary figure legends**

**Supplementary Fig. 1. The impact of PVT1 inhibition on PC cell growth.**

(A) qRT-PCR analysis revealed that endogenous PVT1 was inhibited in BxPC-3 and PANC-1 cells. Data are normalized to si-Con treated cells and GAPDH was used as a loading control. (B) CCK-8 assay was employed to determine the IC50 value of gemcitabine in BxPC-3, PANC-1, and AsPC-1 cells. (C) Cell growth analysis was conducted in BxPC-3 (left panel) and PANC-1 (right panel) cells with normal saline (NS) treatment by using CCK-8 assay on 12 h intervals up to 72 h. (D, E) Cell cycle analyses were performed in BxPC-3 (D) and PANC-1 (E) cells, and normalization of cell numbers at G1-, S-, and G2/M-phase is shown in yellow, blue, and red, respectively. Average values and S.D.s were calculated from triplicate samples.

**Supplementary Fig. 2. Overexpression of *pvt1*-encoded miRNAs is determined in PC cell lines.**

(A, B) qRT-PCR analysis revealed that miR-1204, miR-1207-5p, miR-1207-3p, and miR-1208 were overexpression in BxPC-3 (A) and PANC-1 (B) cells. Data are normalized to scramble-treated cells and U6 snRNA was used as loading control.

**Supplementary Fig. 3. The impact of *pvt1*-encoded miRNAs on PC cell growth.**

(A, B, C) CCK-8 assay was used to investigate the impact of miR-1204 (A), miR-1207-5p (B), and miR-1207-3p (C) on cell growth in BxPC-3 (left panel) and PANC-1 (right panel) cells with NS or gemcitabine treatment by using CCK-8 assay on 12 h intervals up to 72 h.

**Supplementary Fig. 4. The impact of miR-1208 on PC cell growth.**

(A, B) CCK-8 assay was employed to determine the cell growth of PANC-1 (A) and BxPC-3 (B) cells with miR-1208 overexpression upon NS or gemcitabine treatment by using CCK-8 assay on 12 h intervals up to 72 h.

**Supplementary Fig. 5. Overexpression of miR-1207 pair leads to decreased cell numbers at S-phase.**

(A, B) miR-1207-5p miR-1207-3p were overexpressed in BxPC-3 (A) and PANC-1 (B) cells with or without gemcitabine treatment and Cell cycle analyses were conducted. Normalization of cell numbers at G1-, S-, and G2/M-phase is shown in yellow, blue, and red, respectively. Average values and S.D.s were calculated from triplicate samples.

**Supplementary Fig. 6. Gemcitabine promotes the processing of PVT1 in PC cell lines.**

(A, B) The expression of PVT1 (A) and Pri-1207 (B) was evaluated in Capan-1, PCT, MIA PaCa2, Su.86.86, and SW1990 PC cell lines treated with gemcitabine, as shown by qRT-PCR analysis. GAPDH serves as a loading control. (C) The expression levels of mature miR-1207-5p and miR-1207-3p were assessed in Capan-1, PCT, MIA PaCa2, Su.86.86, and SW1990 PC cell lines with gemcitabine treatment. U6 snRNA is used as a loading control.

**Supplementary Fig. 7. Inhibition of miR-1207 pair leads to increased cell numbers at S-phase.**

(A, B) miR-1207-5p miR-1207-3p were inhibited in BxPC-3 (A) and PANC-1 (B) cells with or without gemcitabine treatment and Cell cycle analyses were conducted. Normalization of cell numbers at G1-, S-, and G2/M-phase are shown in yellow, blue, and red, respectively. Average values and S.D.s were calculated from triplicate samples.

**Supplementary Fig. 8. The expression of Drosha and DGCR8 is determined in PC cells with the inhibition of Drosha and DGCR8.**

(A) The endogenous expression of Drosha and DGCR8 was inhibited in BxPC-3 and PANC-1 cells upon specific siRNAs transfection as shown by qRT-PCR analysis. GAPDH served as a loading control, and data were normalized to si-Con-treated cells. (B) The endogenous expression of Drosha (left panel) and DGCR8 (right panel) was inhibited in BxPC-3 and PANC-1 cells upon specific siRNAs transfection as shown by immunoblotting analysis. GAPDH served as a loading control.

**Supplementary Fig. 9. The expression of Drosha and DGCR8 is determined in PC cells with the overexpression of Drosha and DGCR8.**

(A) Drosha or DGCR8 was overexpressed in BxPC-3 and PANC-1 cells as shown by qRT-PCR analysis. GAPDH served as a loading control, and data were normalized to vector-transfected cells. (B) Drosha (left panel) or DGCR8 (right panel) was overexpressed in BxPC-3 and PANC-1 cells as shown by immunoblotting analysis. GAPDH served as a loading control.

**Supplementary Fig. 10. Inhibition of Drosha or DGCR8 leads to increased cell numbers at S-phase.**

(A, B) Drosha and DGCR8 were inhibited in BxPC-3 (A) and PANC-1 (B) cells with or without gemcitabine treatment and Cell cycle analyses were conducted. Normalization of cell numbers at G1-, S-, and G2/M-phase are shown in yellow, blue, and red, respectively. Average values and S.D.s were calculated from triplicate samples.

**Supplementary Fig. 11. Overexpression of Drosha or DGCR8 leads to decreased cell numbers at S-phase.**

Drosha or DGCR8 were overexpressed in AsPC-1 cells with or without gemcitabine treatment and Cell cycle analyses were conducted. Normalization of cell numbers at G1-, S-, and G2/M-phase are shown in yellow, blue, and red, respectively. Average values and S.D.s were calculated from triplicate samples.

**Supplementary Fig. 12. The expression of MYC is determined in PC cells with gemcitabine treatment.**

qRT-PCR analysis was conducted to evaluate the expression of MYC in BxPC-3 and PANC-1 cells upon gemcitabine treatment. GAPDH is used as a loading control, and data are normalized to NS-treated cells.
